# Shredder identity matters: taxon-specific gut microbiota in aquatic shredders drives bacterial diversity during leaf breakdown in streams

**DOI:** 10.1093/ismeco/ycag094

**Published:** 2026-04-10

**Authors:** Pratiksha Acharya, Mourine J Yegon, Luca Zoccarato, Christian Griebler, Simon Vitecek, Katrin Attermeyer

**Affiliations:** WasserCluster Lunz—Biological Station, Dr. Carl Kupelwieser-Prom. 5, Lunz am See 3293, Austria; Department of Functional and Evolutionary Ecology, Unit Limnology, University of Vienna, Djerassiplatz 1, Vienna 1030, Austria; WasserCluster Lunz—Biological Station, Dr. Carl Kupelwieser-Prom. 5, Lunz am See 3293, Austria; Institute for Hydrobiology and Water Management (IHG), University of Natural Resources and Life Sciences, Gregor-Mendel-Straße 33/DG, Vienna 1180, Austria; Institute of Computational Biology, University of Natural Resources and Life Sciences, Muthgasse 18, Vienna 1190, Austria; Core Facility Bioinformatics, University of Natural Resources and Life Sciences, Muthgasse 18, Vienna 1190, Austria; Department of Functional and Evolutionary Ecology, Unit Limnology, University of Vienna, Djerassiplatz 1, Vienna 1030, Austria; WasserCluster Lunz—Biological Station, Dr. Carl Kupelwieser-Prom. 5, Lunz am See 3293, Austria; Institute for Hydrobiology and Water Management (IHG), University of Natural Resources and Life Sciences, Gregor-Mendel-Straße 33/DG, Vienna 1180, Austria; Department of Ecology, University of Innsbruck, Technikerstraße 25, Innsbruck 6020, Austria; WasserCluster Lunz—Biological Station, Dr. Carl Kupelwieser-Prom. 5, Lunz am See 3293, Austria; Department of Functional and Evolutionary Ecology, Unit Limnology, University of Vienna, Djerassiplatz 1, Vienna 1030, Austria

**Keywords:** leaf litter decomposition, gut microbiota, faecal pellets, shredder ontogeny, stream ecosystem

## Abstract

Microbial communities play a key role in organic matter (OM) decomposition and nutrient cycling in headwater streams. Macroinvertebrate shredders enhance these processes by producing large amounts of fine particles from microbe-colonized OM through shredding and egesta, yet how gut and faecal microbiota vary among shredder taxa remains largely unknown. Thus, we examined composition, diversity, and predicted functions of bacterial communities in leaves, guts, and faecal pellets of three Trichoptera shredders (*Allogamus*, *Potamophylax*, and *Sericostoma*) across two larval stages using 16S rRNA metabarcoding. Bacterial community composition differed significantly among the shredder taxa and between sample types, while ontogeny had only a minimal effect. *Sericostoma* showed the lowest diversity but the most distinct microbiota in their gut and faecal pellets, while *Allogamus* and *Potamophylax* hosted more diverse and overlapping bacterial communities. *Carnobacterium* and *Tyzzerella* were specialized bacteria in the shredder guts. Functional predictions showed clear metabolic transitions from aromatic compound degradation in leaves to amino acid and carbohydrate metabolism in guts and continued degradation of plant-derived substrates in faecal pellets, reflecting the shift from environmental to host-associated microbial processes during gut passage. Unique pathways in *Sericostoma* guts, such as palmitate and peptidoglycan biosynthesis, suggest host-specific adaptations. Overall, shredder identity rather than ontogenetic stage was the dominant factor structuring bacterial assemblages and functions, emphasizing the taxon-specific role of shredders as mediators of microbial dynamics, linking microbial structure with ecosystem-level processes in the stream detritus-food-web.

## Graphical Abstract

**Graphical Abstract:**
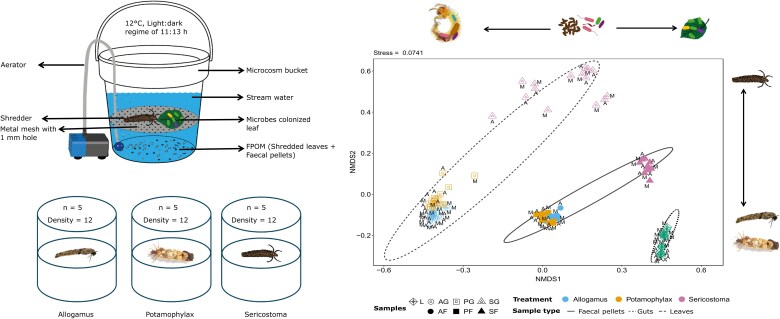

## Introduction

The stream microbiome consists of microorganisms, primarily bacteria but also archaea, fungi, protists, and viruses, distributed across distinct habitats, including the water column, epilithic biofilms on rocks, submerged leaves, suspended organic matter (OM), and streambed sediments. The composition of the stream microbiome is influenced by environment and hydrology, which in turn affect the ecological dynamics depending on the functional roles of the selected community [1–4]. For instance, surface water often harbours Actinobacteria [5], rock-associated biofilms are enriched in Cyanobacteria and Bacteroidetes [1], while submerged leaves are colonized by common leaf decomposers, such as *Pseudomonas, Sphingomonas,* and *Pedobacter* [6, 7]. Since different microbial taxa contribute differently to OM decomposition, nutrient cycling, and energy transfer across trophic levels [8, 9], shifts in bacterial community composition can alter biogeochemical cycles at the ecosystem-level.

Connectivity with surrounding terrestrial environments also contributes to the spatial structuring of stream microbiome through inputs of soil, groundwater, and plant OM [2, 4, 10, 11]. Such inputs are particularly important in headwater streams, where regular leaf litter supply fuels a detritus-based food web [11, 12]. Among the biotic components involved in OM processing, detritivore invertebrates, especially shredders, play a crucial role. They fragment leaf litter, i.e. coarse particulate OM (>1 mm), into fine particulate OM (FPOM <1 mm) via sloppy feeding and egestion of faecal pellets. These FPOM act as microbial hotspots [13], and harbour diverse and specialized bacterial communities that vary depending on particle origin and processing (e.g. fragmented leaf particles vs. faecal pellets) [14]. Shredders themselves are taxonomically diverse, including species within Trichoptera (e.g. Limnephilidae), Amphipoda (e.g. Gammaridae), Plecoptera (e.g. Leuctridae), Coleoptera (e.g. Ptilodactylidae), and certain Diptera larvae (Tipulidae) [15–19], and differ in feeding behaviour [20], mouthpart morphology, digestion efficiency, and habitat preference [21–24]. These traits influence both the rate and pathways of leaf litter breakdown [25], and shape the gut- and faecal pellets-associated microbial communities [13, 26].

Generally, the gut represents a highly dynamic bacterial habitat, influenced by, amongst others, diet, host gut physiology, and ontogenetic stages [17, 27–34]. The gut microbiota have a profound role in health, nutrition, immunity, and even behaviour in vertebrates and invertebrates [29, 35–37]. To date, most invertebrate gut microbiome studies have focused on terrestrial decomposers, such as earthworms, beetles, and termites, or aquatic herbivores (i.e. filter-feeding bivalves and grazers), along with their role in the decomposition of lignocellulose, detoxification of secondary plant metabolites, or synthesis of essential nutrients [27, 31, 38–44]. Here, *Carnobacterium*, *Leucobacter*, *Acinetobacter*, *Vagococcus*, and *Rhodococcus* were commonly reported as gut bacteria in Diptera larvae [45] and other detritivores such as silkworm [44] and green bottle fly larvae [46]. Apart from a handful of studies comparing gut microbiota across insects functional feeding groups [17, 18, 27, 39], there is still a major knowledge gap regarding the community composition of gut microbiota of Trichoptera shredder taxa, which have a prominent role in processing terrestrial OM in headwater streams [22, 23, 34, 47, 48].

In addition to processing ingested OM, shredder gut microbiota can actively influence the structure and composition of bacterial communities in streams once deposited in faecal pellets [14]. During gut passage, OM-associated bacterial community composition changes likely through selective digestion [49–52] or seeding from resident gut microbes [50, 53–55], and this is reflected in the faecal pellet communities. Shredder faecal pellets, an important fraction of in-stream FPOM, thus may facilitate microbial dispersal of gut-associated microbiota into the stream ecosystem [14, 26, 39, 51, 53, 55], and possibly reshape downstream microbial assemblages and OM processing. Specifically, these faecal pellet-associated microbes can remain metabolically active [14, 26, 53, 56], continuing detrital decomposition and thereby contribute to the release of dissolved carbon, nitrogen, and phosphorus [13, 38, 57, 58]. Furthermore, they may interact with streambed biofilms to form complex microbial consortia, which are critical for nutrient retention and energy flow [9]. However, the relative persistence of leaf-associated bacteria versus the seeding of gut-associated bacteria on faecal pellets during gut passage remains poorly understood. Clarifying these bacterial linkages is essential for understanding the broader ecological role of shredders, particularly how their gut microbiota influence downstream bacterial dynamics and ecosystem functioning.

It has been demonstrated that the structure of gut microbiomes can change during different developmental life stages (= ontogenetic stages) across a wide range of animal hosts, including insects, amphibians, and mammals [30, 32, 34, 59]. These changes are often driven by dietary transitions, developmental modifications in digestive physiology, such as pH, oxygen level, independent of diet, and the maturation of the immune system [17, 27, 37, 60]. Also, freshwater invertebrates across functional feeding groups have different gut physiology [61–63], as well as gut-associated microbiota [18, 39, 64] and functions during different ontogenetic stages [65]. Many freshwater shredders change their feeding preferences and digestive capabilities during their transition from one to another larval stage, which may influence the structure of their gut microbial communities [32, 33]. However, information about the shift in gut microbiota of freshwater shredders across ontogenetic stages is still missing.

In this study, we aim to elucidate how shredder-mediated leaf litter decomposition influences the particle-attached bacterial communities and how they contribute to the stream microbiome. Specifically, we investigate (i) how gut passage modifies the diversity, composition, and functional potential of bacterial communities on faecal pellets compared to those on leaf litter, (ii) to what extent the bacterial community in faecal pellets is derived from leaf-associated bacteria and gut bacteria, and (iii) how the diversity and composition of gut-associated bacterial communities vary across ontogenetic stages. To address these questions, we performed two sequential microcosm experiments where we incubated individually three of the most abundant Trichoptera shredder taxa—*Allogamus auricollis* (Order: Trichoptera, Family: Limnephilidae), *Potamophylax latipennis* (Order: Trichoptera, Family: Limnephilidae), and *Sericostoma flavicorne*. (Order: Trichoptera, Family: Sericostomatidae) [23, 48, 59, 66] with alder leaves in early April and late May. We investigated the bacterial communities on the leaves, in the gut of each taxon at different developmental stages, and the collected faecal pellets using DNA and RNA metabarcoding.

## Materials and methods

### Experimental microcosm setup

We conducted feeding experiments in a climate-controlled chamber (12°C; 11:13 h light:dark) with alder leaves and three different shredder taxa (*Allogamus*, *Potamophylax*, *Sericostoma*). The first experiment began in early April and the second in late May 2024 (~2 months interval) to target different larval stages of the shredder taxa. The experiments were performed with five replicates for each shredder treatment containing 12 individuals and alder leaves (dry mass: 1.0 ± 0.003 g). Five controls containing only leaves were set up to assess baseline changes in bacterial community composition during the 5-day incubation period.

Each microcosm consisted of a white food-grade plastic bucket (1100 ml volume and 122 mm bottom diameter) with a steel mesh (pore size 1 mm) suspended at a height of 4.5–5 cm from the bottom to allow shredder-derived FPOM to pass through. The shredders were fed with conditioned leaves for 5 days. Leaf and shredder gut microbiome samples were collected at the beginning (= natural baseline animals) and end (= experimental animals). After the 5-day incubation period, we transferred each larva from each treatment to a single food-grade plastic cup (Polylactic acid or polylactide—PLA) filled with 20 ml of 0.7 μm-filtered stream water and left them to evacuate their gut contents for 24 h [19]. In this way, we made sure that we only collected faecal pellets and no shredded leaf particles, and that larvae had empty guts before subsequent analyses. We collected the faecal pellets in the plastic cup using a Pasteur pipette after collecting and storing the experimental animals at −70°C until further processing (for more details of microcosms setup see [13]).

### Preparation of conditioned alder leaves

We collected naturally fallen leaves from alder trees (*Alnus glutinosa*) around Lunz am See, Austria, in autumn 2023, dried and stored them at room temperature. Prior to microbial colonization, we cut leaves into 12-mm diameter circular discs, avoiding major leaf veins. For the oxic microbial colonization, we submerged the leaf discs in aerated, freshly collected stream water from Oberer Seebach (OSB; 47°51′ N, 15°04′ E) at 12°C in the dark for 1 week.

### Insect’s sampling and preparation

A day before each feeding experiment, we collected comparably sized larvae of *Allogamus*, *Potamophylax*, and *Sericostoma* (Trichoptera) from Oberer Seebach (OSB; 47°51′ N, 15°04′ E) and brought them to the laboratory. We determined larval stages of targeted animals by measuring the head capsule width (HCW). In April, *Allogamus* was in early V instars (HCW = 1.57 ± 0.05 mm) [48], *Potamophylax* in IV instars (HCW = 2.18 ± 0.03 mm) [66], and *Sericostoma* in early IV instars (HCW = 1.96 ± 0.04 mm) [59, 67]. In May, the targeted larvae were mostly in the late instars; *Allogamus* late V instars (HCW = 1.68 ± 0.02) [48], *Potamophylax* V instars (HCW = 2.23 ± 0.03 mm) [66], and *Sericostoma* in late IV instars (HCW = 2.03 ± 0.05 mm) [59, 67]. Here, the two sampling months reflect the natural, species-specific timing of life cycle stages. Although differences in HCW were sometimes small, they represent ecologically meaningful ontogenetic transitions shown to influence feeding behaviour and leaf processing [68]. Each larva was placed in a separate PLA cup (volume: 4 cl), with filtered stream water (0.7 μm, 450°C, 4 h pre-combusted Whatmann GF/F filters) at 12°C and starved for 24 h before the start of the experiment to allow evacuation of their guts [19]. On the next day, we randomly sub-sampled 70 individuals from these freshly collected wild populations per taxon per experiment to have a natural gut microbiota baseline.

### Gut dissection

We dissected guts from 12 shredder larvae per microcosm at the end of the experiment, as well as from 12 individuals per taxon initially collected for the natural baseline. After removing larval cases, each shredder larva was dissected aseptically in a petri dish filled with 5 ml of 95% ethanol under a stereomicroscope. An incision using sterile forceps and a scalpel was made longitudinally along the body wall, and the entire gut, from the clitellum to anus, was removed and placed in a 1.5 ml Eppendorf tube filled with 400 μl RNA/DNA shield (Zymo Research Europe GmbH, Freiburg, Germany) for the preservation of genetic material. The samples were then stored in the refrigerator at 4°C until DNA/RNA extraction.

### DNA/RNA extraction

For DNA/RNA extraction, we pooled gut content and faecal pellets, respectively, from 12 larvae per microcosm. However, our tests revealed that this amount was too much for DNA extraction of *Potamophylax* gut microorganisms, and we thus pooled the guts from five larvae per microcosm in case of *Potamophylax*. Overall, we had five replicates for natural baseline animals, experimental animals, and faecal pellets from each shredder taxon for each experiment. We extracted total genomic DNA and RNA from all sample types, including leaves [*n* = 10 with 2 (initial leaves + control leaf treatments)^*^5 replicates], shredder guts [*n* = 30 with 2 (natural baseline + experimental animals)^*^3 shredder taxa^*^5 replicates] and faecal pellets produced by each shredder taxon (*n* = 15 with 3 shredder taxa^*^5 replicates) for each experiment using the ZymoBIOMICS DNA/RNA Miniprep Kit (Zymo Research Europe GmbH, Freiburg, Germany) following the manufacturers’ protocol (leaves were first cut into smaller pieces with a sterile scalpel). To stabilize extracted RNA, we converted them into complementary DNA (cDNA) using the Reverse Transcription Kit (Qiagen, Hilden, Germany) following the manufacturers’ protocol. Afterwards, concentrations were quantified fluorometrically with the Qubit™ 1X dsDNA HS Assay Kit for DNA and the Qubit™ ssDNA Assay Kit for cDNA using an Invitrogen Qubit 4.0 Fluorometer (Thermo Fisher Scientific). The replicates were the same for the cDNA datasets related to the leaves and faecal pellets, yet we do not have a cDNA dataset for the shredders’ gut samples because we were not able to recover a measurable amount of material.

### Amplicon sequencing

We sent the extracted total DNA and cDNA to the Joint Microbiome Facility of the University of Vienna for amplification and sequencing the V4 region of the 16S rRNA gene. The primer pair, 515F (GTG YCA GCM GCC GCG GTA A) and 806R (GGA CTA CNV GGG TWT CTA AT) [69, 70] was used for amplification; barcoding and adapter ligation were achieved in a second step as described in [71] for sequencing on an Illumina MiSeq platform with 600-cycle v3 chemistry (2 × 300 bp paired-end reads). Raw sequencing data were processed using a FASTQ workflow (Basespace, Illumina) with default settings, and sorted into unique amplicon sequence variants (ASVs) using the DADA2 pipeline [72] in R [73] using the recommended workflow. The representative ASVs generated were subsequently merged and classified using the SILVA r138.1 database using default parameters [74], and taxonomy assigned using the assignTaxonomy() function in DADA2 with a confidence threshold of 0.05 for prokaryotic 16S rRNA gene amplicons.

### Data filtering

We kept the DNA and cDNA datasets separately, and processed (filtering and stats) them identically. Before statistical analysis, we removed ASVs affiliated to Eukaryota, Archaea, mitochondria, chloroplasts (indicative of insect tissues and ingested plant material), and unknown phyla, as the sampling designs were not optimized for capturing these organisms. To reduce the effect of rare taxa on subsequent analyses, we excluded ASVs found in fewer than three samples, as well as singleton and doubleton, except for calculations of the Chao1 index. We also removed the samples with library sizes <1500 reads prior to downstream analyses to ensure reliable community estimates. Hereafter, the leaves and gut samples represent the combined dataset from the beginning and end of the feeding experiment. We combined both datasets because we found no significant differences in the bacterial community composition of gut samples from natural baseline and experimental animals. Here, we calculated bias-corrected omega squared (ω^2^) from the permutational multivariate analysis of variance (PERMANOVA) sums of squares [75] as an estimate of the proportion of distance-based multivariate variance explained by each factor (PERMANOVA; *F*_(1,48)_ = 1.565, *R*^2^ = 0.333, *P* = .187; ω^2^ = 0.007, i.e. negligible effect size). For leaf samples, although differences were statistically significant, the proportion of variance explained was small (*F*_(1,18)_ = 3.345, *R*^2^ = 0.156, *P* = .022; ω^2^ = 0.028, i.e. below the threshold for medium effect). Given the low effect sizes, combining the beginning and end samples to increase the number of replicates, and thus strengthen the statistical analysis, was justified. Finally, we combined ASV read abundances, taxonomy, and sample metadata into a single dataset (function *phyloseq*; *phyloseq* package) [76] in R for subsequent analyses. All data analyses and visualizations were done in R 4.5.0 version [73].

### Data visualization and statistical analyses

To visualize the bacterial taxonomic composition in the samples, we generated bar plots (function *plot_microshades*; *microshades* package, function *comp_barplot*; *microViz* package) [77, 78], aggregating ASVs (function *tax_glom*; *phyloseq* package) at the phylum, order, or genus level.

To assess bacterial diversity during and after gut transit, we calculated Shannon index from the rarified (sequencing depth = 2400 for DNA samples and sequencing depth = 450 for cDNA samples) ASV tables (function *estimate_richness; phyloseq* package). We used the unprocessed dataset for Chao1 calculation. We then tested the sample groups’ differences in alpha diversity metrics (Shannon and Chao1) by running a linear model (function *lm*; *stats* package) where shredder taxa, sample type (gut vs. faecal pellets), month, and their interactions were used as predictors. For each model, we calculated the effect size (function *effectsize*; *effectsize* package) [79] to identify the strongest influencing fixed factor. Goodness of fit (normality of residuals, homogeneity of variance, and collinearity) was checked using graphical procedures. Afterwards, we calculated the estimated marginal means values of Shannon and Chao1 indices (function *emmeans; emmeans* package) [80] and report adjusted *p*-values after using a multivariate *t*-distribution approach for pairwise comparisons among the groups (function *pairs*; *emmeans* package) [80].

To test compositional differences of bacterial communities between leaves, guts, and faecal pellets, we calculated beta diversity using Hellinger-transformed ASV abundances (function *decostand*; *vegan* package). We visualized bacterial community composition using non-metric multidimensional scaling (NMDS; function *ordinate*; *phyloseq* package) based on Bray–Curtis dissimilarities of transformed ASVs abundances (function *vegdist*; *vegan* package). We evaluated the goodness of fit of the NMDS by the stress value, with values above 0.2 indicating unreliable ordinations (https://jkzorz.github.io/2019/06/06/NMDS.html). We tested for differences in community composition (beta diversity) between sample types (leaves, gut, and faecal pellets) using PERMANOVA (function *adonis2; vegan* package) on the transformed ASVs. The assumption of homogeneity of within-group dispersion was tested (function *betadisper*; *vegan* package) and was fulfilled for all groups. Further, we tested the differences in composition of gut-associated bacterial communities across shredder taxa and across their ontogenetic stages using analysis of similarities (Analysis of Similarities; ANOSIM; function *anosim*; *vegan* package).

To examine the numbers/proportions of shared (core) and unique bacterial taxa among leaves, guts, and faecal pellets across shredder taxa, we visualized taxonomic overlaps using Venn diagrams (function *ggvenn*; *ggvenn* pacakage).

We predicted the functional potential of bacterial communities using PICRUSt2 (https://github.com/picrust/picrust2; Version 2.2.0) [81], focusing on the METACYC pathways, hereafter referred to as pathways, for downstream analyses. We used pathway abundance tables as the input for defining core pathways (present in all samples of a given group) and unique pathways (>90% prevalence within a group and <50% in others).

We performed a differential abundance analysis using DESeq2 [82], applying negative binomial models to both the raw ASV count (aggregated at genus level) and the pathways abundance tables. Significance was tested using Wald tests, and we adjusted the *P*-values for multiple comparisons using the Benjamini–Hochberg false discovery rate correction [83]. We visualized the differentially abundant taxa and predicted pathways across leaves, guts, and faecal pellets for each shredder taxon using heatmaps (function pheatmap; pheatmap package). For the taxa, we selected and visualized the top 100 differential genera as they represented >80% of the read counts, discarding the unclassified genus group. Similarly, we focused on and showed pathways with a log fold change >2.5.

## Results

### Composition of bacterial communities in leaves, shredder guts, and faecal pellets

**Figure 1:**
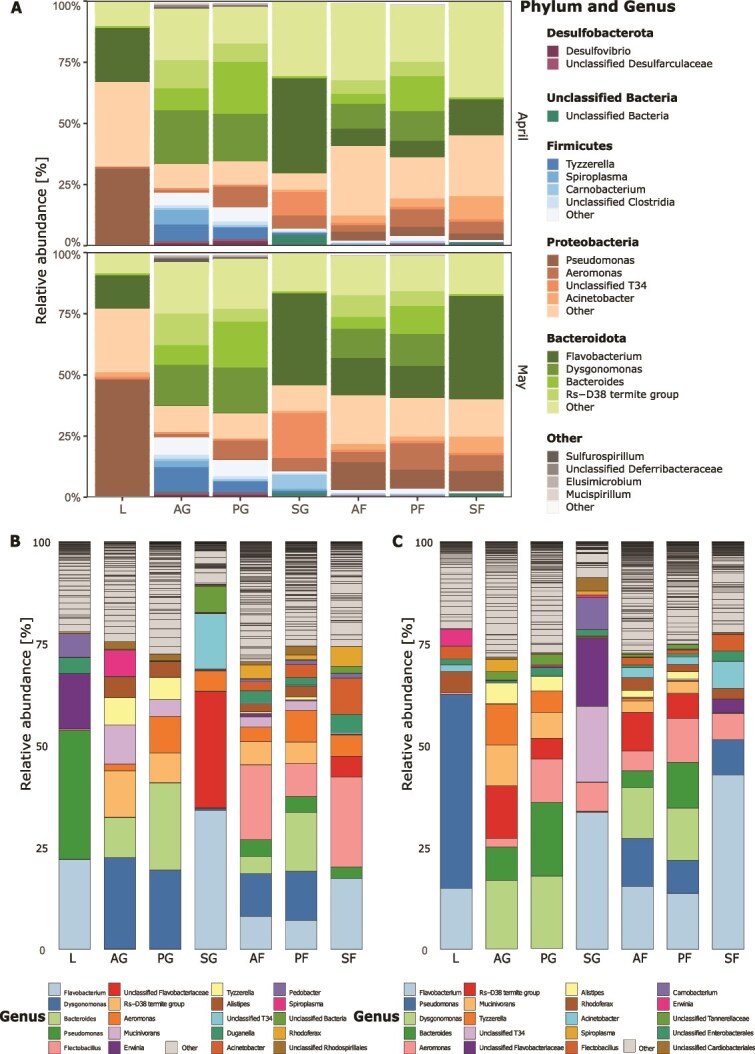
Taxonomic composition and relative abundance of individual taxonomic groups associated with leaves, guts of each shredder taxon, and faecal pellets produced by each shredder taxon during the feeding experiment in the months of April and May based on DNA dataset. In Fig. 1A, each phylum is indicated by a different colour, and each genus is indicated by a different shade of the colour. Only the five most abundant phyla and four most abundant genera across phyla level for each treatment are shown whereas the remaining phyla and genera are grouped as “Other.” In Fig. 1B (April) and 1C (May), each genus is represented by a distinct colour; the top 20 genera by relative abundance are shown, and all remaining genera are grouped as “Other.” L, leaves; AG, Allogamus gut; AF, Allogamus faecal pellets; PG, Potamophylax gut; PF, Potamophylax faecal pellets; SG, Sericostoma gut; SF, Sericostoma faecal pellets.

The taxonomic composition of bacterial communities at different levels (phylum and genus) varied among leaves, guts, and faecal pellets of different shredders, but only small differences were detected (Mantel test; *r* = 0.45, *P* = .001) when comparing DNA (Fig. 1) and cDNA results (Supplementary Fig. S1). Hereafter, we will always refer to the DNA dataset outcomes unless explicitly stated. Based on relative abundances of ASVs across months and sample type (excluding leaves), the most abundant bacterial phyla were Bacteroidota followed by Proteobacteria, Firmicutes, and Desulfobacterota. Proteobacteria was the most abundant phylum in leaves (Fig. 1A, Supplementary Table S1). Within Firmicutes, *Tyzzerella, Spiroplasma,* and *Carnobacterium* were the most abundant genera in samples from guts of different shredders (Fig. 1A, Supplementary Fig. S1). Additionally, cDNA data showed that *Bacteroides* and *Rs-D38 termite group* were abundant in the faecal pellets of shredders (Supplementary Fig. S1, Supplementary Table S2).

### Diversity of bacterial communities in leaves, shredder guts, and faecal pellets

**Figure 2:**
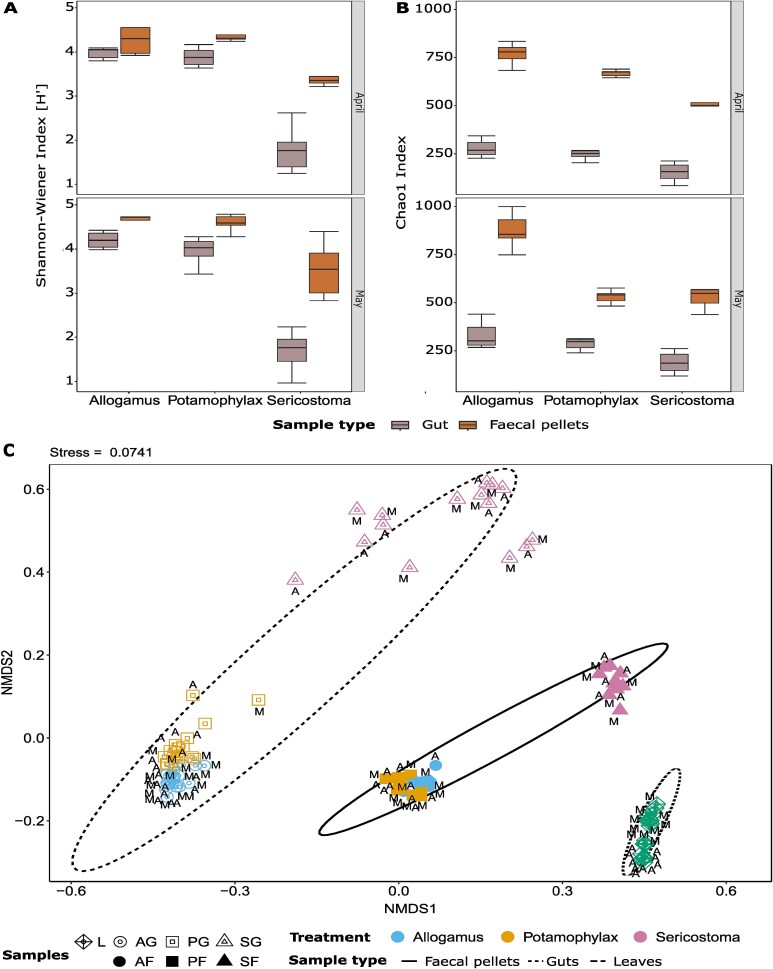
Alpha and beta diversity of bacterial communities in different samples. Shannon (A) and Chao1 (B) metrics for samples collected in the months of April and May. Non-metric dimensional scaling (NMDS) showing the clustering of normalized and Hellinger-transformed 16S sequencing data based on Bray–Curtis distances of bacterial communities associated with leaves, gut, and faecal pellets of three different shredder taxa (C). Here, the bacterial communities in L, leaves; AG, Allogamus gut; AF, Allogamus faecal pellets; PG, Potamophylax gut; PF, Potamophylax faecal pellets; SG, Sericostoma gut; SF, Sericostoma faecal pellets are shown by different symbols, and four colours represent the three shredder taxa and the leaves. A, April and M, May distinguish between the two different months where samples were collected from the feeding experiments. The dotted, solid, and semi-dotted line of ellipses (99% confidence interval) encircle the shredder guts’, faecal pellets, and leaves collected during the experiment, respectively. The two-dimensional NMDS solution had a stress value of 0.0741, indicating a good representation of multivariate distances in reduced space.

Comparison of the bacterial alpha diversity (Shannon, Chao1) between the sample types showed that the gut and faecal pellets of *Sericostoma* had the lowest bacterial diversity, followed by guts and faecal pellets of *Allogamus* and *Potamophylax* (Supplementary Table S3, Fig. 2A and B). Shredder taxa had the strongest effect on Shannon bacterial diversity, while sample type (gut vs. faecal pellets) had strongest effect on Chao1 bacterial diversity (Table 1). Among the three shredder taxa, *Sericostoma* had a negative significant effect on bacterial diversity compared to the reference shredder taxa “*Allogamus*” (linear model: Estimate = −0.913, *P* < .05 for Shannon index and −29.471, <0.05 for Chao1 index; see Supplementary Table S4 for all effect sizes). *Potamophylax* had no significant effect on both diversity indices (Supplementary Table S4, S5). In addition, there were significant differences in alpha diversity between samples from April and May for the Shannon index (Table 1, Supplementary Table S4, S5).

**Table 1:** Effects of shredder taxa (*Allogamus*, *Potamophylax*, *Sericostoma*.), sample type (guts, faecal pellets), month as a proxy for ontogenetic stage, and their interactions, on bacterial diversity of samples collected during feeding experiment, based on linear model analysis.

Factors	St. coefficient	SS	df	*F*-value	*P*-value	St. coefficient	SS	df	*F*-value	*P*-value
Sample type	0.03	0.24	1	2.04	0.16	0.88	1 585 833	1	513.21	**<0.05**
Shredder taxa	0.28	3.22	2	13.79	**<0.05**	0.55	260 357	2	42.13	**<0.05**
Month	0.06	0.51	1	4.40	**<0.05**	0.03	7437	1	2.41	0.13
Sample type:Shredder taxa	0.25	2.79	2	11.97	**<0.05**	0.13	32 582	2	5.27	**<0.05**
Sample type:Month	0.01	0.09	1	0.76	0.39	0.03	7353	1	2.38	0.13
Shredder taxa:Month	0.01	0.12	2	0.49	0.61	0.19	50 761	2	8.21	**<0.05**
Sample type:Shredder taxa:Month	0.01	0.05	2	0.22	0.81	0.18	48 305	2	7.82	**<0.05**
Residuals		8.16	70				216 302	70		

St, Standardized; SS, sum of squares; df, degrees of freedom. Note: Statistically significant p-values (< 0.05) are shown in bold.

The NMDS ordination and PERMANOVA analysis demonstrated that the bacterial communities associated with leaves, guts, and faecal pellets were clearly distinct from one another (*F*_(2,98)_ = 42.349, *R*^2^ = 0.454, *P* < .001). The separation along NMDS1 corresponds to the differences in the different sample types and NMDS2 separated the bacterial communities associated with the gut and faecal pellets of *Sericostoma* from those of the other two shredder taxa (Fig. 2C). We found a significant difference in gut-associated bacterial communities across shredder taxa (ANOSIM; *R* = 0.7775, *P* < .001) but not across months (ANOSIM; *R* = 0.03222, *P* = .113).

### Core and unique bacterial communities and the predicted pathways between leaves, guts, and faecal pellets

**Figure 3:**
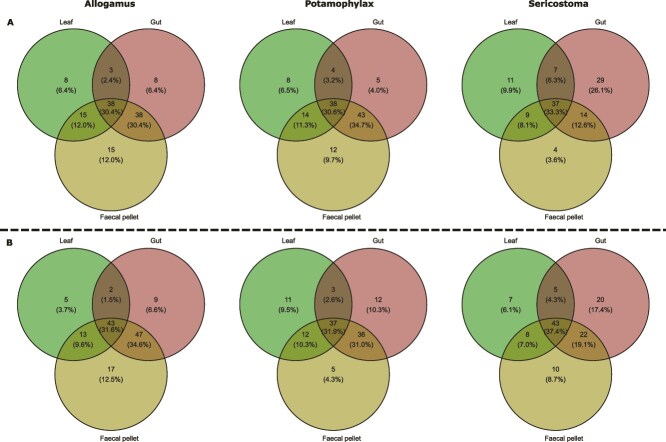
Venn diagrams showing the number and percentages of shared and unique ASVs in leaves, guts, and faecal pellets of Allogamus, Potamophylax, and Sericostoma at family level for the months of April (A) and May (B).

The Venn diagrams showed that a large proportion of taxa are shared across leaves, guts, and faecal pellets for all three shredder taxa and both months (Fig. 3). In April, a comparison of bacterial communities of the resource (alder leaves) with the guts and faecal pellets at family level showed 30.4%, 30.6% and 34.2% shared (core) ASVs for *Allogamus*, *Potamophylax*, and *Sericostoma*, respectively (Fig. 3). Furthermore, in April, we observed a similar proportion of shared ASVs between the gut and faecal pellets with 30.4% for *Allogamus* and 34.7% for *Potamophylax*. *Sericostoma*, however, showed a lower proportion of shared ASVs between its gut and faecal pellets, with 12.6%, but a larger proportion of unique gut ASVs (26.1%) compared to *Allogamus* (6.4%) and *Potamophylax* (4.0%). The proportions of shared and unique ASVs among leaves, guts, and faecal pellets for each shredder taxon in May were broadly similar to those observed in April (Fig. 3).

**Figure 4:**
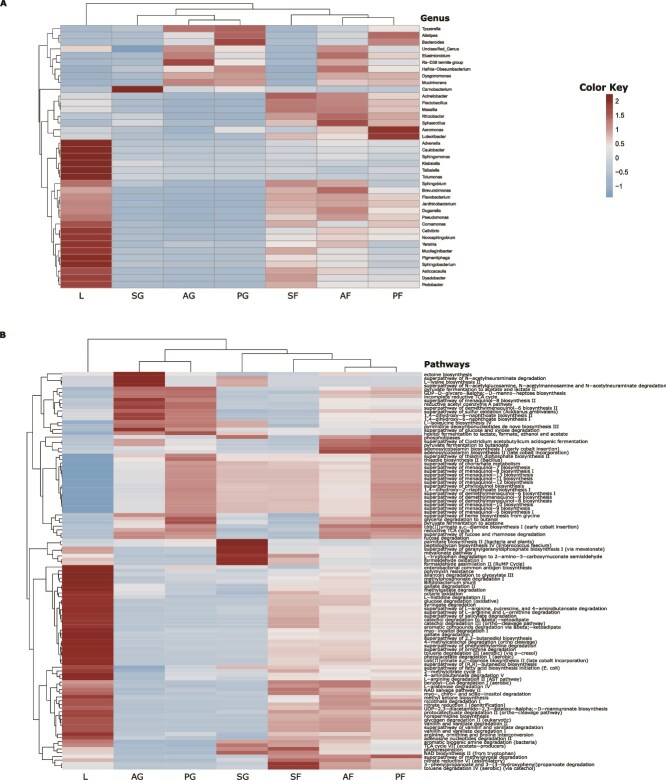
Hierarchical heat map of differentially abundant taxa (A) and pathways (B), showing the log2 transformed abundant values for leaves as well as the guts, and faecal pellets of each shredder taxa. The differentially abundant individual taxa and pathways across the leaves (L), guts (AG, Allogamus gut; PG, Potamophylax gut; SG, Sericostoma gut), and faecal pellets of each shredder taxa (AF, Allogamus faecal pellets; PF, Potamophylax faecal pellets; SF, Sericostoma faecal pellets) are shown horizontally. The colour key reflects normalized log-transformed counts with skyblue for lower abundance and red colour for higher abundance.

Analysis at genus level revealed that ASVs occurring uniquely in gut samples corresponded to genera *Dysgonomonas, Mucinivorans, Carnobacterium, Tyzzerella,* and *Rs−D38 termite group* (Fig. 4A, Supplementary Fig. S1). Based on prevalence percentage, superpathway of N-acetylglucosamine, N-acetylmannosamine, and N-acetylneuraminate degradation, lysine synthesis, and pyruvate fermentation were unique pathways in the guts, while degradation of toluene, propanoate, and catechol were unique pathways in faecal pellets of shredders (see Supplementary Table S6).

### Differentially abundant genera and pathways in leaves, shredder guts, and faecal pellets

The hierarchical clustering of differentially abundant genera and differential pathways showed coherent pattern among sample type, i.e. leaves, guts, and faecal pellets of each shredder taxon (Fig. 4). *Pseudomonas*, *Flavobacterium*, *Pedobacter*, *Novosphingobium,* and *Sphingobium* were differentially abundant genera in the leaves (Fig. 4A, see Supplementary Fig. S2A for cDNA dataset) while genera such as *Carnobacterium*, and *Tyzzerella* were enriched in the guts, and *Flavobacterium*, *Pseudomonas*, *Bacteroides*, *Dysgonomonas*, *Acinetobacter*, *Elusimicrobium Rs−D38 termite group, Mucinivorans* in faecal pellets (Fig. 4A). Furthermore, cDNA data showed *Acinetobacter, Duganella, Flectobacillus, Polaromonas,* and *Undibacterium* were active and enriched in faecal pellets of *Sericostoma* while Bacteroides*, Elusimicrobium,* and *Tyzzerell* were enriched in faecal pellets of *Allogamus* and *Potamophylax* (Supplementary Fig. S2A).

These taxon-specific enrichments were consistent with different functional pathways. Leaves showed an enrichment in the degradation of aromatic compounds, toluene, catechol, syringate, gallate, and vanillin, and vanillate (Fig. 4B, Supplementary Table S7, see Supplementary Fig. S2B, Supplementary Table S8 for cDNA dataset). L-lysine biosynthesis II and superpathway of N-acetylneuraminate degradation were enriched in guts of *Allogamus* and *Sericostoma* (Fig. 4B, Supplementary Table S7), and nitrate reduction VI (assimilatory), degradation of 2-nitrobenzoate, and L-tryptophan were abundant in faecal pellets of *Allogamus* and *Sericostoma* (Fig. 4B, Supplementary Fig. S2B, Supplementary Tables S7 and S8). Furthermore, superpathway of demethylmenaquinol-6 biosynthesis II and menaquinol-8 biosynthesis II were enriched in the guts of *Allogamus* and *Potamophylax* (Fig. 4B, Supplementary Table S7). Interestingly, palmitate and peptidoglycan biosynthesis were differential pathways in the gut of *Sericostoma* only (Fig. 4B, Supplementary Table S7).

## Discussion

By comparing leaf biofilm bacteria with gut- and faecal pellet-associated microbiota of different shredder taxa, we assessed how shredder-mediated leaf litter decomposition shapes particle-attached bacterial communities, and thereby influences the structure, diversity, and ecological functions of the stream microbiome. Our findings revealed significant differences in bacterial communities and predicted functions associated with leaf particles, shredder guts, and faecal pellets. These differences were dependent on Trichoptera shredder identity, indicating that the composition and activity of stream bacterial communities are influenced by which shredders are present. In contrast, ontogeny (using sampling months as a proxy, validated by HCW) had only a minimal effect. Together, these findings showed that shredder identity rather than ontogenetic stage is a primary factor shaping bacterial assemblages in the gut and in shredder-produced faecal pellets. This underscores shredders as key drivers of the stream microbiome, not only through leaf fragmentation but also through the gut-mediated microbial filtration and inoculation.

### Shredder identity driving the bacterial community structure

Among the three Trichoptera shredders we studied, *Sericostoma* guts and faecal pellets had the lowest bacterial diversity (Fig. 2A) and different community composition compared to the guts and faecal pellets of *Allogamus* and *Potamophylax* (Fig. 2C). Other insect studies have also shown species-specific differences in gut microbiomes [17, 27, 29, 31, 43, 84]. So far, most of the studies assessing insect gut microbiomes focused on differences between different feeding types [17, 37, 39] with fewer studies addressing differences within the same feeding types [43, 84, 85]. For instance, a study from Lange *et al.* has advocated that the host species can have unique internal gut filtering process and physio-chemical conditions in their gut, such as gut pH, oxygen levels, and immune system activity, which select for specific microbial communities. This aligns with our results, suggesting that there are more species-specific traits than just the food the species feed on that determines its gut microbiome [27, 43, 84].

The consistently lower bacterial diversity in *Sericostoma* suggests that its gut may be more selective for specific bacteria, possibly due to digestive physiology, enzyme activity, or narrower feeding preferences, allowing only certain bacterial groups to persist. In contrast, *Allogamus* and *Potamophylax* hosted more diverse microbiota, implying a less restrictive gut habitat. Yet, we did not test actual gut microenvironment parameters across the taxa. These insects mostly feed on leaf fragments and detritus [22, 34, 48, 59]. However, *Allogamus* is a facultative filter-feeder/grazer and shredder [22, 48], and potentially encountering more diverse microbial sources while switching feeding modes [86]. Furthermore, *Allogamus* and *Potamophylax* typically inhabit uppermost sediment layers while *Sericostoma* dwells deeper (up to 1 m) and feeds on fine detritus and mosses along with leaf litter [23, 34, 59, 87]. These differences in habitat and feeding preferences likely expose shredders to distinct environmental microbial pools, and consequently, have contributed to the establishment of species-specific core gut microbiomes that persisted throughout the experiment.

Taxonomic closeness of *Allogamus* and *Potamophylax* (Limnephilidae) as opposed to *Sericostoma* (Sericostomatidae) suggests their evolutionary differences, which might have an effect on their gut physiology and microbial filtering. This likely explains the distinct gut microbiota in *Sericostoma* as compared to the other two taxa, although all taxa belonging to the same functional feeding group. Noteworthy, these taxonomic patterns were further evident in the predicted functional profiles (Fig. 4B). For instance, *Sericostoma* gut microbiota showed several unique pathways, including palmitate and peptidoglycan biosynthesis, that can be linked to differentially abundant taxa—*Carnobacterium* (Phyla: Firmicutes) [88]. Together with the scarcity of *Carnobacterium* in *Sericostoma* faecal samples, our evidence supports that the gut microbiome of *Sericostoma* is highly specialized and strongly linked to their host physiology, as shown for other organisms [23, 43, 63, 85].

To sum up, the interspecific variability in both taxonomic composition and predicted functions among the shredder gut microbiota suggests that shredders not only contribute to the physical fragmentation of leaves, but to the functional recycling of microbial communities within stream detritus. By introducing taxon-specific microbial and functional signatures via faecal pellets into the streams, shredders possibly influence downstream microbial activity, nutrient cycling, and food-web dynamics, thereby acting as biotic connectors that bridge microbial processing across habitats [20, 21].

### Gut passage functions as a filter and a vector of microbial dispersal

Food transit through the gut has a dual effect on bacterial communities. First, it acts as a filter, selectively promoting or inhibiting certain bacterial taxa already present on ingested material [26, 49, 50, 53, 89]. Second, it acts as a microbial dispersal vector, introducing gut-derived bacteria into faecal pellets, and re-distributing ingested leaf-associated microbes back into the environment through egestion [26, 50, 51, 53, 55]. Lower bacterial diversity in guts compared to faecal pellets, as indicated by Shannon and Chao1 indices in our data, shows that faecal pellets contain a higher number of different bacterial taxa than the gut. This pattern suggests that the gut functions as a selective environment, retaining only certain taxa. Faecal pellets, in contrast, contain both gut-resident bacteria and additional leaf-associated bacteria that pass through the gut but do not establish there, resulting in higher diversity [26, 51, 53, 89]. Therefore, gut passage contributes to overall bacterial diversity in the stream microbiome, and this could be crucial for the resilience of stream functions and health. Furthermore, our findings align with the idea that faecal pellets contribute to the seeding of downstream microbial assemblages and influence the processing of OM beyond the site of initial ingestion [49, 51, 53].

We found a larger proportion of shared bacterial taxa between guts and faecal pellets (12%–35%) than between leaves and faecal pellets (8%–15%, see Fig. 3), indicating that gut microbiota exert a stronger influence on the faecal pellets-associated bacterial composition than the leaf material itself. Venn diagram (Fig. 3) showing certain proportion of unique bacterial taxa in faecal pellets further supported that gut passage facilitates the enrichment of taxa not previously detected in the ingested material. Yet, these unique taxa in faecal pellets cannot be exclusively attributed to gut passage, as larval cases may also serve as favourable microhabitats, and likely contributed to faecal pellets-associated microbial inoculation during the 24-h gut-emptying period. cDNA data further showed substantial numbers of active and unique bacterial taxa in both leaves and faecal pellets (Supplementary Fig. S2A). Several taxa, particularly *Bacteroides*, *Elusimicrobium,* and *Tyzzerella,* were differentially abundant in guts of *Allogamus* and *Potamophylax* (see Fig. 4A) and also present as active members in faecal pellets (see Supplementary Fig. S2A). This indicates that the living gut-resident bacteria were introduced to faecal pellets [41, 52–55], and possibly further contribute to egested particles processing. Enrichment of *Acinetobacter* and *Pseudomonas* in faecal pellets, both known for their role in aromatic compound degradation, such as polysaccharides, might point to the inoculation of these functions into the stream and thereby accelerating OM turnover [90–92]. Furthermore, leaf-associated communities, such as several variants of *Flavobacterium*, *Pseudomonas*, and *Sphingobium,* were consistently abundant in leaves as well as present in faecal samples. This implies that these bacteria possibly continue to degrade plant-derived polymers even after gut passage [93, 94]. Additionally, active genera including *Aeromonas*, *Flavobacterium*, and *Pseudomonas* (Supplementary Fig. S2A) in faecal pellets of different shredders likely facilitate the leaf litter decomposition by reducing either nitrate or nitrite and producing N_2_O, as these are known bacteria involved in the nitrogen cycle [95]. Together, these findings suggest that shredders not only passively redistribute microbes through their guts but also actively shape functional roles of bacteria associated with faecal pellets, which continue to further contribute to detrital degradation.

We further showed clear metabolic transitions from aromatic compound degradation in leaves to a dominance of amino acid and carbohydrate metabolism in guts and faecal pellets (Fig. 4B). Our results thus suggest the active involvement of bacteria in the breakdown of lignin-derived substrates and polyphenols on leaves [44, 90, 92]. Gut microbiota were enriched in biosynthesis and host-substrate metabolism, such as L-lysine biosynthesis, N-acetylneuraminate degradation (mucin), and menaquinol biosynthesis, indicating that gut bacteria specialize in rapid processing of nitrogen-rich and easily digestible substrates. This likely reflects shifts in bacterial metabolism favourable for the hosts’ digestive processes. Furthermore, the enrichment of specialized pathways, including tryptophan degradation and aromatic compound meta-cleavage pathways in faecal pellets-associated with active bacteria (see Supplementary Fig. S2B), may indicate the continuity of bacterial degradation of plant-derived compounds even after egestion. These findings emphasize the pivotal role of shredders in enhancing the stream’s microbiome functions by introducing faecal pellets enriched with active and functionally specialized bacteria, and thereby supporting complex metabolic processes downstream.

### Minimal ontogenetic influence

Studies of terrestrial insects and other aquatic macroinvertebrates showed that gut microbiota and their functions differed between ontogenetic stages on their life history [37, 45, 65, 96]. In our study, ontogenetic stage had a small but significant effect on Shannon diversity, but overall bacterial community structure remained stable across months. This suggests that, once established, shredder gut microbiota remains relatively consistent, despite slight changes in diet, microhabitat, and environmental microbial inputs. A similar stability of the gut microbiome during later larval instars was observed in other holometabolous insects [30, 84]. However, it is possible that substantial shifts may occur during later life stages, particularly during pupation and metamorphosis, as seen in other terrestrial insects [32, 33, 97]. For instance, honey bee larval guts are dominated by Firmicutes and those of adults by Proteobacteria, including probiotics taxa, suggesting the importance of specific bacteria at each life stage for improving host’s health [96, 97]. Moreover, a seasonal analysis of silkworm gut revealed changes in bacterial community composition between summer and winter, as well as between early and late instars, which is linked to the silkworm’s development and has implications for lignocellulose degradation [44]. However, we did not find clear ontogenetic influence on the gut microbiota or associated predicted pathways in our shredders. Therefore, future studies could trace the influence of ontogeny on gut microbiota of aquatic shredders across the early-to-late larval stages.

## Conclusion

Overall, our findings show that shredder identity is a key factor shaping the stream microbiome. Different shredder taxa host distinct gut- and faecal pellet-associated bacterial communities, likely driven by taxon-specific traits such as host physiology, gut morphology, feeding preferences, and microhabitat. These taxonomic differences are supported by predicted functions, indicating faecal pellets from different shredders have distinct impacts on bacterial assemblages and the potential functions of the stream microbiome. In particular, *Sericostoma* showed a highly selective and specialized gut microbiome, emphasizing that not all shredders contribute equally to bacterial diversity or decomposition pathways. Gut passage appeared as a major driver of bacteria redistribution, where shredder guts act as both a filter, selectively promoting or inhibiting bacterial taxa and potential functions, and a microbial dispersal vector, redistributing gut-and leaf-associated bacteria into the environment via faecal pellets. In contrast, ontogenetic stage (as measured by months) had a minimal effect on bacterial community structure, indicating a strong founder effect and relative stability of gut microbiota once established. While metagenomic and metatranscriptomic approaches are essential to resolve active metabolic networks and microbial interactions during leaf litter decomposition, our results demonstrated that shredders are important mediators linking bacterial structure with ecosystem-level processes in detritus-based stream food-webs. Understanding these host-bacteria linkages is critical for predicting how environmental change or biodiversity loss might affect shredder communities and their gut microbiota, thereby affecting overall stream ecosystem functioning and health.

## Supplementary Material

Supplementary_revised_ycag094

## Data Availability

The sequence datasets generated and analyzed during the current study are available in the Sequence Read Archive (SRA) of the National Center for Biotechnology Information (NCBI) under BioProject ID—PRJNA1321319.
