# Supplementary material for: Shredder identity matters: taxon-specific gut microbiota in aquatic shredders drives bacterial diversity during leaf breakdown in streams
Source: ISME Commun. 2026 Apr 10;6(1):ycag094. doi: 10.1093/ismeco/ycag094 (PMC13174403; doi:10.1093/ismeco/ycag094)
Supplement: Supplementary_revised_ycag094 [file supplementary_revised_ycag094.docx]

**Short title, “Shredder identity matters”**

**Main title, “Shredder identity matters: Taxon-specific gut microbiota in aquatic shredders drives bacterial diversity during leaf breakdown in streams”**

Pratiksha Acharya^1,2^*, Mourine J. Yegon^1,3^, Luca Zoccarato^5,6^, Christian Griebler^2^, Simon Vitecek^1,3,4^, Katrin Attermeyer^1,2^

^1^ WasserCluster Lunz – Biological Station, Dr. Carl Kupelwieser-Prom. 5, 3293 Lunz am See, Austria

^2^ Department of Functional and Evolutionary Ecology, Unit Limnology, University of Vienna, Djerassiplatz 1, 1030 Vienna, Austria

^3^ Institute for Hydrobiology and Water Management (IHG), University of Natural Resources and Life Sciences, Gregor-Mendel-Straße 33/DG, 1180 Vienna, Austria

^4^ Department of Ecology, University of Innsbruck, Technikerstraße 25, 6020 Innsbruck, Austria

^5^Institute of Computational Biology, University of Natural Resources and Life Sciences, Muthgasse 18
1190 Vienna, Austria

^6^Core Facility Bioinformatics, University of Natural Resources and Life Sciences, Muthgasse 18, 1190, Vienna, Austria

*Corresponding author email: [Pratiksha.Acharya@wcl.ac.at](mailto:Pratiksha.Acharya@wcl.ac.at)

Results:

After removing all the undesirable taxa, we used 23,509 reads to identify 1370 taxa with a median of 8964 per DNA sample and 32,262 reads to identify 1370 taxa with a median of 5620 per cDNA sample. After we filtered out low abundance reads, 1248 taxa remained for DNA samples and 1085 taxa for cDNA samples, which we agglomerated into 20 phyla, 77 order, 141 family, and 239 genera for DNA samples and 19 phyla, 61 order, 125 family, and 208 genera for cDNA samples. Comparing the bacterial community composition between DNA and cDNA, we found that there were only small differences. Thus, we mostly focus on the DNA here if not explicitly stated.

**Table S1.** Taxonomic classification of bacterial communities with more than 2 % relative abundances present in different samples are shown here. Here data includes whole genomic DNA.

| **Month** | **Sample_type** | **Treatment** | **Phylum** | **Order** | **Family** | **Genus** | **Relative abundance**  **[%]** |
| --- | --- | --- | --- | --- | --- | --- | --- |
| April | Leaf | Leaves | Proteobacteria | Pseudomonadales | Pseudomonadaceae | Pseudomonas | 31.63 |
|  |  |  | Bacteroidota | Flavobacteriales | Flavobacteriaceae | Flavobacterium | 22.08 |
|  |  |  | Proteobacteria | Enterobacterales | Erwiniaceae | Erwinia | 13.58 |
|  |  |  | Bacteroidota | Sphingobacteriales | Sphingobacteriaceae | Pedobacter | 5.89 |
|  |  |  | Proteobacteria | Burkholderiales | Oxalobacteraceae | Duganella | 3.88 |
|  | Gut | Allogamus | Bacteroidota | Bacteroidales | Dysgonomonadaceae | Dysgonomonas | 22.35 |
|  |  |  | Bacteroidota | Bacteroidales | Williamwhitmaniaceae | Rs-D38 termite group | 11.41 |
|  |  |  | Bacteroidota | Bacteroidales | Bacteroidaceae | Bacteroides | 9.85 |
|  |  |  | Bacteroidota | Bacteroidales | Rikenellaceae | Mucinivorans | 9.54 |
|  |  |  | Firmicutes | Lachnospirales | Lachnospiraceae | Tyzzerella | 6.62 |
|  |  |  | Firmicutes | Entomoplasmatales | Spiroplasmataceae | Spiroplasma | 6.44 |
|  |  |  | Bacteroidota | Bacteroidales | Rikenellaceae | Alistipes | 5.2 |
|  |  |  | Bacteroidota | Bacteroidales | Tannerellaceae | Unclassified Tannerellaceae | 2.02 |
|  |  | Potamophylax | Bacteroidota | Bacteroidales | Bacteroidaceae | Bacteroides | 21.38 |
|  |  |  | Bacteroidota | Bacteroidales | Dysgonomonadaceae | Dysgonomonas | 19.4 |
|  |  |  | Proteobacteria | Enterobacterales | Aeromonadaceae | Aeromonas | 8.99 |
|  |  |  | Bacteroidota | Bacteroidales | Williamwhitmaniaceae | Rs-D38 termite group | 7.25 |
|  |  |  | Firmicutes | Lachnospirales | Lachnospiraceae | Tyzzerella | 5.4 |
|  |  |  | Bacteroidota | Bacteroidales | Rikenellaceae | Mucinivorans | 4.07 |
|  |  |  | Bacteroidota | Bacteroidales | Rikenellaceae | Alistipes | 3.85 |
|  |  |  | Bacteroidota | Bacteroidales | Unclassified Bacteroidales | Unclassified Bacteroidales | 2.21 |
|  |  |  | Desulfobacterota | Desulfovibrionales | Desulfovibrionaceae | Desulfovibrio | 2.17 |
|  |  |  | Proteobacteria | Burkholderiales | Rhodocyclaceae | Propionivibrio | 2.17 |
|  |  | Sericostoma | Bacteroidota | Flavobacteriales | Flavobacteriaceae | Flavobacterium | 34.11 |
|  |  |  | Bacteroidota | Flavobacteriales | Flavobacteriaceae | Unclassified Flavobacteriaceae | 28.5 |
|  |  |  | Proteobacteria | Burkholderiales | T34 | Unclassified T34 | 13.54 |
|  |  |  | Unclassified Bacteria | Unclassified Bacteria | Unclassified Bacteria | Unclassified Bacteria | 6.37 |
|  |  |  | Proteobacteria | Enterobacterales | Aeromonadaceae | Aeromonas | 4.96 |
|  |  |  | Proteobacteria | Cardiobacteriales | Unclassified Cardiobacteriales | Unclassified Cardiobacteriales | 2.52 |
|  | Faecal pellets | Allogamus | Bacteroidota | Cytophagales | Spirosomaceae | Flectobacillus | 18.39 |
|  |  |  | Bacteroidota | Bacteroidales | Dysgonomonadaceae | Dysgonomonas | 10.43 |
|  |  |  | Bacteroidota | Flavobacteriales | Flavobacteriaceae | Flavobacterium | 8.13 |
|  |  |  | Bacteroidota | Bacteroidales | Williamwhitmaniaceae | Rs-D38 termite group | 5.7 |
|  |  |  | Bacteroidota | Bacteroidales | Bacteroidaceae | Bacteroides | 4.23 |
|  |  |  | Proteobacteria | Pseudomonadales | Pseudomonadaceae | Pseudomonas | 4.12 |
|  |  |  | Proteobacteria | Enterobacterales | Aeromonadaceae | Aeromonas | 3.49 |
|  |  |  | Proteobacteria | Burkholderiales | Comamonadaceae | Rhodoferax | 3.28 |
|  |  |  | Proteobacteria | Burkholderiales | Oxalobacteraceae | Duganella | 2.92 |
|  |  |  | Bacteroidota | Bacteroidales | Rikenellaceae | Mucinivorans | 2.57 |
|  |  |  | Proteobacteria | Pseudomonadales | Moraxellaceae | Acinetobacter | 2.19 |
|  |  |  | Proteobacteria | Burkholderiales | Methylophilaceae | Methylotenera | 2.12 |
|  |  |  | Proteobacteria | Caulobacterales | Caulobacteraceae | Brevundimonas | 2.03 |
|  |  | Potamophylax | Bacteroidota | Bacteroidales | Bacteroidaceae | Bacteroides | 14.44 |
|  |  |  | Bacteroidota | Bacteroidales | Dysgonomonadaceae | Dysgonomonas | 12.07 |
|  |  |  | Bacteroidota | Cytophagales | Spirosomaceae | Flectobacillus | 7.99 |
|  |  |  | Proteobacteria | Enterobacterales | Aeromonadaceae | Aeromonas | 7.73 |
|  |  |  | Bacteroidota | Flavobacteriales | Flavobacteriaceae | Flavobacterium | 7.09 |
|  |  |  | Bacteroidota | Bacteroidales | Williamwhitmaniaceae | Rs-D38 termite group | 5.33 |
|  |  |  | Proteobacteria | Pseudomonadales | Pseudomonadaceae | Pseudomonas | 3.92 |
|  |  |  | Proteobacteria | Pseudomonadales | Moraxellaceae | Acinetobacter | 3.3 |
|  |  |  | Bacteroidota | Bacteroidales | Rikenellaceae | Alistipes | 2.59 |
|  |  |  | Bacteroidota | Bacteroidales | Rikenellaceae | Mucinivorans | 2.40 |
|  |  |  | Proteobacteria | Rhodospirillales | Unclassified Rhodospirillales | Unclassified Rhodospirillales | 2.17 |
|  |  | Sericostoma | Bacteroidota | Cytophagales | Spirosomaceae | Flectobacillus | 22.03 |
|  |  |  | Bacteroidota | Flavobacteriales | Flavobacteriaceae | Flavobacterium | 17.42 |
|  |  |  | Proteobacteria | Pseudomonadales | Moraxellaceae | Acinetobacter | 8.92 |
|  |  |  | Proteobacteria | Enterobacterales | Aeromonadaceae | Aeromonas | 5.23 |
|  |  |  | Bacteroidota | Flavobacteriales | Flavobacteriaceae | Unclassified Flavobacteriaceae | 5.13 |
|  |  |  | Proteobacteria | Burkholderiales | Comamonadaceae | Rhodoferax | 4.95 |
|  |  |  | Proteobacteria | Burkholderiales | Oxalobacteraceae | Duganella | 4.45 |
|  |  |  | Bacteroidota | Cytophagales | Spirosomaceae | Arcicella | 2.98 |
|  |  |  | Proteobacteria | Pseudomonadales | Pseudomonadaceae | Pseudomonas | 2.8 |
| May | Leaf | Leaves | Proteobacteria | Pseudomonadales | Pseudomonadaceae | Pseudomonas | 47.59 |
|  |  |  | Bacteroidota | Flavobacteriales | Flavobacteriaceae | Flavobacterium | 14.92 |
|  |  |  | Proteobacteria | Burkholderiales | Comamonadaceae | Rhodoferax | 5.01 |
|  |  |  | Proteobacteria | Enterobacterales | Erwiniaceae | Erwinia | 4.26 |
|  |  |  | Bacteroidota | Cytophagales | Spirosomaceae | Flectobacillus | 3.16 |
|  | Gut | Allogamus | Bacteroidota | Bacteroidales | Dysgonomonadaceae | Dysgonomonas | 16.83 |
|  |  |  | Bacteroidota | Bacteroidales | Williamwhitmaniaceae | Rs-D38 termite group | 12.86 |
|  |  |  | Firmicutes | Lachnospirales | Lachnospiraceae | Tyzzerella | 10.09 |
|  |  |  | Bacteroidota | Bacteroidales | Rikenellaceae | Mucinivorans | 9.98 |
|  |  |  | Bacteroidota | Bacteroidales | Bacteroidaceae | Bacteroides | 8.16 |
|  |  |  | Bacteroidota | Bacteroidales | Rikenellaceae | Alistipes | 5.04 |
|  |  |  | Firmicutes | Entomoplasmatales | Spiroplasmataceae | Spiroplasma | 3.07 |
|  |  |  | Proteobacteria | Burkholderiales | Burkholderiaceae | Unclassified Burkholderiaceae | 2.66 |
|  |  |  | Proteobacteria | Enterobacterales | Aeromonadaceae | Aeromonas | 2.13 |
|  |  | Potamophylax | Bacteroidota | Bacteroidales | Bacteroidaceae | Bacteroides | 18.01 |
|  |  |  | Bacteroidota | Bacteroidales | Dysgonomonadaceae | Dysgonomonas | 17.75 |
|  |  |  | Proteobacteria | Enterobacterales | Aeromonadaceae | Aeromonas | 10.7 |
|  |  |  | Bacteroidota | Bacteroidales | Rikenellaceae | Mucinivorans | 6.35 |
|  |  |  | Firmicutes | Lachnospirales | Lachnospiraceae | Tyzzerella | 5.15 |
|  |  |  | Bacteroidota | Bacteroidales | Williamwhitmaniaceae | Rs-D38 termite group | 5.11 |
|  |  |  | Bacteroidota | Bacteroidales | Rikenellaceae | Alistipes | 3.67 |
|  |  |  | Bacteroidota | Bacteroidales | Tannerellaceae | Unclassified Tannerellaceae | 2.67 |
|  |  |  | Bacteroidota | Bacteroidales | Dysgonomonadaceae | Unclassified Dysgonomonadaceae | 2.11 |
|  |  |  | Proteobacteria | Enterobacterales | Unclassified Enterobacterales | Unclassified Enterobacterales | 2.07 |
|  |  | Sericostoma | Bacteroidota | Flavobacteriales | Flavobacteriaceae | Flavobacterium | 33.64 |
|  |  |  | Proteobacteria | Burkholderiales | T34 | Unclassified T34 | 18.57 |
|  |  |  | Bacteroidota | Flavobacteriales | Flavobacteriaceae | Unclassified Flavobacteriaceae | 16.72 |
|  |  |  | Firmicutes | Lactobacillales | Carnobacteriaceae | Carnobacterium | 7.85 |
|  |  |  | Proteobacteria | Enterobacterales | Aeromonadaceae | Aeromonas | 7.06 |
|  |  |  | Proteobacteria | Cardiobacteriales | Unclassified Cardiobacteriales | Unclassified Cardiobacteriales | 3.27 |
|  |  |  | Unclassified Bacteria | Unclassified Bacteria | Unclassified Bacteria | Unclassified Bacteria | 2.36 |
|  |  |  | Proteobacteria | Rickettsiales | Rickettsiaceae | Rickettsia | 2.11 |
|  | Faecal pellets | Allogamus | Bacteroidota | Flavobacteriales | Flavobacteriaceae | Flavobacterium | 15.5 |
|  |  |  | Bacteroidota | Bacteroidales | Dysgonomonadaceae | Dysgonomonas | 12.46 |
|  |  |  | Proteobacteria | Pseudomonadales | Pseudomonadaceae | Pseudomonas | 11.68 |
|  |  |  | Bacteroidota | Bacteroidales | Williamwhitmaniaceae | Rs-D38 termite group | 9.53 |
|  |  |  | Proteobacteria | Enterobacterales | Aeromonadaceae | Aeromonas | 4.8 |
|  |  |  | Bacteroidota | Bacteroidales | Bacteroidaceae | Bacteroides | 4.18 |
|  |  |  | Proteobacteria | Burkholderiales | Comamonadaceae | Rhodoferax | 3.1 |
|  |  |  | Bacteroidota | Bacteroidales | Rikenellaceae | Mucinivorans | 2.74 |
|  |  |  | Proteobacteria | Pseudomonadales | Moraxellaceae | Acinetobacter | 2.35 |
|  |  |  | Proteobacteria | Burkholderiales | Oxalobacteraceae | Massilia | 2.08 |
|  |  | Potamophylax | Bacteroidota | Flavobacteriales | Flavobacteriaceae | Flavobacterium | 13.75 |
|  |  |  | Bacteroidota | Bacteroidales | Dysgonomonadaceae | Dysgonomonas | 12.86 |
|  |  |  | Bacteroidota | Bacteroidales | Bacteroidaceae | Bacteroides | 11.21 |
|  |  |  | Proteobacteria | Enterobacterales | Aeromonadaceae | Aeromonas | 10.79 |
|  |  |  | Proteobacteria | Pseudomonadales | Pseudomonadaceae | Pseudomonas | 8.06 |
|  |  |  | Bacteroidota | Bacteroidales | Williamwhitmaniaceae | Rs-D38 termite group | 6.11 |
|  |  |  | Bacteroidota | Bacteroidales | Rikenellaceae | Mucinivorans | 2.86 |
|  |  | Sericostoma | Bacteroidota | Flavobacteriales | Flavobacteriaceae | Flavobacterium | 42.79 |
|  |  |  | Proteobacteria | Pseudomonadales | Pseudomonadaceae | Pseudomonas | 8.59 |
|  |  |  | Proteobacteria | Pseudomonadales | Moraxellaceae | Acinetobacter | 6.63 |
|  |  |  | Proteobacteria | Enterobacterales | Aeromonadaceae | Aeromonas | 6.53 |
|  |  |  | Bacteroidota | Cytophagales | Spirosomaceae | Flectobacillus | 4.15 |
|  |  |  | Bacteroidota | Flavobacteriales | Flavobacteriaceae | Unclassified Flavobacteriaceae | 3.23 |
|  |  |  | Proteobacteria | Burkholderiales | Comamonadaceae | Rhodoferax | 2.64 |
|  |  |  | Proteobacteria | Enterobacterales | Unclassified Enterobacterales | Unclassified Enterobacterales | 2.48 |
|  |  |  | Bacteroidota | Chitinophagales | Unclassified Chitinophagales | Unclassified Chitinophagales | 2.28 |
|  |  |  | Unclassified Bacteria | Unclassified Bacteria | Unclassified Bacteria | Unclassified Bacteria | 2.08 |
|  |  |  | Proteobacteria | Burkholderiales | Oxalobacteraceae | Massilia | 2.02 |

**Table S2.** Taxonomic classification of active bacterial communities with more than 2 % relative abundances present in different samples are shown here. Here data includes complementary DNA.

| **Month** | **Sample_type** | **Treatment** | **Phylum** | **Order** | **Family** | **Genus** | **Relative**  **Abundance**  **[%]** |
| --- | --- | --- | --- | --- | --- | --- | --- |
| April | Leaves | Leaves | Proteobacteria | Pseudomonadales | Pseudomonadaceae | Pseudomonas | 31.91 |
|  |  |  | Bacteroidota | Flavobacteriales | Flavobacteriaceae | Flavobacterium | 16.92 |
|  |  |  | Proteobacteria | Burkholderiales | Comamonadaceae | Inhella | 6.05 |
|  |  |  | Proteobacteria | Enterobacterales | Erwiniaceae | Erwinia | 5.22 |
|  |  |  | Proteobacteria | Burkholderiales | Comamonadaceae | Rhodoferax | 4.22 |
|  |  |  | Proteobacteria | Burkholderiales | Oxalobacteraceae | Duganella | 3.49 |
|  |  |  | Proteobacteria | Pseudomonadales | Cellvibrionaceae | Cellvibrio | 2.43 |
|  |  |  | Proteobacteria | Sphingomonadales | Sphingomonadaceae | Sphingomonas | 2.38 |
|  | Faecal pellets | Allogamus | Bacteroidota | Flavobacteriales | Flavobacteriaceae | Flavobacterium | 21.05 |
|  |  |  | Bacteroidota | Cytophagales | Spirosomaceae | Flectobacillus | 6.27 |
|  |  |  | Proteobacteria | Burkholderiales | Comamonadaceae | Rhodoferax | 5.76 |
|  |  |  | Bacteroidota | Bacteroidales | Williamwhitmaniaceae | Rs-D38 termite group | 5.49 |
|  |  |  | Proteobacteria | Pseudomonadales | Pseudomonadaceae | Pseudomonas | 5.00 |
|  |  |  | Proteobacteria | Burkholderiales | Comamonadaceae | Unclassified Comamonadaceae | 4.26 |
|  |  |  | Proteobacteria | Burkholderiales | Oxalobacteraceae | Duganella | 3.83 |
|  |  |  | Proteobacteria | Burkholderiales | Comamonadaceae | Roseateles | 2.96 |
|  |  |  | Proteobacteria | Enterobacterales | Aeromonadaceae | Aeromonas | 2.39 |
|  |  |  | Bacteroidota | Bacteroidales | Bacteroidaceae | Bacteroides | 2.32 |
|  |  |  | Bacteroidota | Bacteroidales | Dysgonomonadaceae | Dysgonomonas | 2.28 |
|  |  | Potamophylax | Bacteroidota | Flavobacteriales | Flavobacteriaceae | Flavobacterium | 15.30 |
|  |  |  | Proteobacteria | Enterobacterales | Aeromonadaceae | Aeromonas | 7.54 |
|  |  |  | Bacteroidota | Bacteroidales | Bacteroidaceae | Bacteroides | 6.80 |
|  |  |  | Bacteroidota | Bacteroidales | Williamwhitmaniaceae | Rs-D38 termite group | 6.21 |
|  |  |  | Proteobacteria | Pseudomonadales | Pseudomonadaceae | Pseudomonas | 5.79 |
|  |  |  | Proteobacteria | Burkholderiales | Oxalobacteraceae | Duganella | 4.44 |
|  |  |  | Bacteroidota | Cytophagales | Spirosomaceae | Flectobacillus | 4.17 |
|  |  |  | Firmicutes | Lachnospirales | Lachnospiraceae | Tyzzerella | 3.94 |
|  |  |  | Proteobacteria | Rhodospirillales | Unclassified Rhodospirillales | Unclassified Rhodospirillales | 2.84 |
|  |  |  | Bacteroidota | Bacteroidales | Dysgonomonadaceae | Dysgonomonas | 2.67 |
|  |  |  | Proteobacteria | Burkholderiales | Comamonadaceae | Rhodoferax | 2.51 |
|  |  |  | Bacteroidota | Bacteroidales | Rikenellaceae | Alistipes | 2.07 |
|  |  | Sericostoma | Bacteroidota | Flavobacteriales | Flavobacteriaceae | Flavobacterium | 22.74 |
|  |  |  | Proteobacteria | Burkholderiales | Oxalobacteraceae | Duganella | 10.45 |
|  |  |  | Proteobacteria | Burkholderiales | Comamonadaceae | Rhodoferax | 10.10 |
|  |  |  | Bacteroidota | Cytophagales | Spirosomaceae | Flectobacillus | 7.48 |
|  |  |  | Proteobacteria | Enterobacterales | Aeromonadaceae | Aeromonas | 4.70 |
|  |  |  | Proteobacteria | Pseudomonadales | Pseudomonadaceae | Pseudomonas | 4.65 |
|  |  |  | Proteobacteria | Burkholderiales | Comamonadaceae | Polaromonas | 3.83 |
|  |  |  | Proteobacteria | Burkholderiales | Oxalobacteraceae | Undibacterium | 3.32 |
|  |  |  | Proteobacteria | Burkholderiales | Comamonadaceae | Roseateles | 3.02 |
|  |  |  | Proteobacteria | Burkholderiales | Comamonadaceae | Unclassified Comamonadaceae | 2.94 |
|  |  |  | Proteobacteria | Burkholderiales | Oxalobacteraceae | Janthinobacterium | 2.48 |
|  |  |  | Bacteroidota | Flavobacteriales | Flavobacteriaceae | Unclassified Flavobacteriaceae | 2.11 |
| May | Leaves |  | Proteobacteria | Pseudomonadales | Pseudomonadaceae | Pseudomonas | 43.14 |
|  |  |  | Proteobacteria | Burkholderiales | Comamonadaceae | Rhodoferax | 9.30 |
|  |  |  | Bacteroidota | Flavobacteriales | Flavobacteriaceae | Flavobacterium | 7.69 |
|  |  |  | Proteobacteria | Burkholderiales | Oxalobacteraceae | Duganella | 5.01 |
|  |  |  | Bacteroidota | Cytophagales | Spirosomaceae | Flectobacillus | 2.76 |
|  |  |  | Proteobacteria | Burkholderiales | Comamonadaceae | Unclassified Comamonadaceae | 2.33 |
|  |  |  | Proteobacteria | Sphingomonadales | Sphingomonadaceae | Sphingomonas | 2.21 |
|  |  |  | Proteobacteria | Enterobacterales | Erwiniaceae | Erwinia | 2.18 |
|  | Faecal pellets | Allogamus | Bacteroidota | Flavobacteriales | Flavobacteriaceae | Flavobacterium | 10.40 |
|  |  |  | Proteobacteria | Pseudomonadales | Pseudomonadaceae | Pseudomonas | 9.55 |
|  |  |  | Bacteroidota | Bacteroidales | Williamwhitmaniaceae | Rs-D38 termite group | 8.29 |
|  |  |  | Proteobacteria | Burkholderiales | Comamonadaceae | Rhodoferax | 6.66 |
|  |  |  | Bacteroidota | Bacteroidales | Dysgonomonadaceae | Dysgonomonas | 4.29 |
|  |  |  | Proteobacteria | Burkholderiales | Oxalobacteraceae | Massilia | 3.74 |
|  |  |  | Proteobacteria | Enterobacterales | Aeromonadaceae | Aeromonas | 3.09 |
|  |  |  | Proteobacteria | Burkholderiales | Comamonadaceae | Roseateles | 2.93 |
|  |  |  | Proteobacteria | Burkholderiales | Comamonadaceae | Unclassified Comamonadaceae | 2.75 |
|  |  |  | Bacteroidota | Bacteroidales | Bacteroidaceae | Bacteroides | 2.58 |
|  |  |  | Proteobacteria | Burkholderiales | Oxalobacteraceae | Duganella | 2.54 |
|  |  |  | Bacteroidota | Sphingobacteriales | env.OPS 17 | Unclassified env.OPS 17 | 2.30 |
|  |  | Potamophylax | Proteobacteria | Pseudomonadales | Pseudomonadaceae | Pseudomonas | 12.83 |
|  |  |  | Bacteroidota | Flavobacteriales | Flavobacteriaceae | Flavobacterium | 11.75 |
|  |  |  | Proteobacteria | Enterobacterales | Aeromonadaceae | Aeromonas | 11.00 |
|  |  |  | Bacteroidota | Bacteroidales | Bacteroidaceae | Bacteroides | 6.29 |
|  |  |  | Bacteroidota | Bacteroidales | Williamwhitmaniaceae | Rs-D38 termite group | 6.17 |
|  |  |  | Proteobacteria | Burkholderiales | Comamonadaceae | Rhodoferax | 4.62 |
|  |  |  | Bacteroidota | Bacteroidales | Dysgonomonadaceae | Dysgonomonas | 3.59 |
|  |  |  | Proteobacteria | Burkholderiales | Oxalobacteraceae | Duganella | 2.90 |
|  |  |  | Proteobacteria | Burkholderiales | Oxalobacteraceae | Massilia | 2.21 |
|  |  |  | Firmicutes | Lachnospirales | Lachnospiraceae | Tyzzerella | 2.09 |
|  |  |  | Proteobacteria | Burkholderiales | Comamonadaceae | Roseateles | 2.00 |
|  |  | Sericostoma | Bacteroidota | Flavobacteriales | Flavobacteriaceae | Flavobacterium | 23.46 |
|  |  |  | Proteobacteria | Pseudomonadales | Pseudomonadaceae | Pseudomonas | 12.66 |
|  |  |  | Proteobacteria | Burkholderiales | Comamonadaceae | Rhodoferax | 7.56 |
|  |  |  | Proteobacteria | Burkholderiales | Oxalobacteraceae | Massilia | 6.13 |
|  |  |  | Proteobacteria | Enterobacterales | Aeromonadaceae | Aeromonas | 5.60 |
|  |  |  | Bacteroidota | Flavobacteriales | Flavobacteriaceae | Unclassified Flavobacteriaceae | 5.28 |
|  |  |  | Proteobacteria | Burkholderiales | Comamonadaceae | Unclassified Comamonadaceae | 3.50 |
|  |  |  | Bacteroidota | Cytophagales | Spirosomaceae | Flectobacillus | 3.46 |
|  |  |  | Proteobacteria | Burkholderiales | Oxalobacteraceae | Duganella | 2.84 |
|  |  |  | Proteobacteria | Burkholderiales | Comamonadaceae | Roseateles | 2.70 |

**Table S3.** Alpha diversity indices are based on rarefaction method for different samples collected during feeding experiment. Mean and standard deviation for species richness (Observed), Chao1 and Shannon diversity based on whole genomic DNA dataset are shown. Abbreviations; L: Leaves, AG: *Allogamous* gut, PG: *Potamophylax* gut, SG: *Sericostoma* gut, AF: *Allogamus* Faecal pellets, PF: *Potamophylax* Faecal pellets and SF: *Sericostoma* Faecal pellets

| **Samples** | **Sample_type** | **Treatment** | **Month** | **Observed** | **Chao1** | **Shannon** |
| --- | --- | --- | --- | --- | --- | --- |
| AF | Faecal pellets | Allogamus | April | 306.4±36.85 | 768.93±58.29 | 4.26±0.30 |
| AF | Faecal pellets | Allogamus | May | 332.2±24.52 | 874.08±94.97 | 4.71±0.16 |
| PF | Faecal pellets | Potamophylax | April | 303±30.30 | 668.32±17.68 | 4.39±0.19 |
| PF | Faecal pellets | Potamophylax | May | 304.6±18.30 | 531.95±36.37 | 4.58±0.20 |
| SF | Faecal pellets | Sericostoma | April | 212.2±10.52 | 492.90±74.90 | 3.35±0.10 |
| SF | Faecal pellets | Sericostoma | May | 233.2±37.32 | 524.45±55.93 | 3.53±0.64 |
| AG | Gut | Allogamus | April | 162.44±16.78 | 284.44±58.96 | 3.99±0.12 |
| AG | Gut | Allogamus | May | 194.6±16.95 | 328.43±61.12 | 4.21±0.17 |
| PG | Gut | Potamophylax | April | 149.2±21.68 | 254.20±40.02 | 3.76±0.43 |
| PG | Gut | Potamophylax | May | 175.33±13.70 | 293.51±44.84 | 3.92±0.39 |
| SG | Gut | Sericostoma | April | 66.28±33.68 | 154.38±49.59 | 1.76±0.48 |
| SG | Gut | Sericostoma | May | 59.5±20.70 | 189.66±53.54 | 1.68±0.42 |
| L | Leaves | Leaves | April | 196.3±10.33 | 418.72±75.24 | 3.55±0.17 |
| L | Leaves | Leaves | May | 190.5±14.62 | 382.81±57.01 | 3.59±0.27 |

**Table S4.** Results of the linear model testing the effects of shredder taxa (treatment = *Allogamus*, *Potamophylax*. and *Sericostoma*.), sample type (Gut vs Faecal pellets), month and their interaction on bacterial diversity. The model structures are H′ ~ Sample_type * Treatment * Month and Chao1 ~ Sample_type * Treatment * Month for Shannon and Chao metrics respectively. Abbreviations: SE, standard error; H′: Shannon–Wiener index, Chao1: Chao index. Data used here was whole genomic DNA dataset.

|  | | **Shannon** |  |  |  | | **Chao1** |  |  |  |
| --- | --- | --- | --- | --- | --- | --- | --- | --- | --- | --- |
| **Factors** | **Estimate** | | **Std. Error** | **t value** | **Pr(>\|t\|)** | **Estimate** | | **Std. Error** | **t value** | **Pr(>\|t\|)** |
| Sample_typeGut | -0.272 | | 0.190 | -1.428 | 0.158 | 291.591 | | 12.871 | 22.654 | **<0.05** |
| TreatmentPotamophylax | 0.127 | | 0.216 | 0.588 | 0.559 | -143.574 | | 15.893 | -9.034 | 0.316 |
| TreatmentSericostoma | -0.913 | | 0.216 | -4.226 | **<0.05** | -29.471 | | 15.634 | -1.885 | **<0.05** |
| MonthMay | 0.453 | | 0.216 | 2.098 | **<0.05** | 19.818 | | 12.774 | 1.551 | 0.096 |
| Sample_typeGut:TreatmentPotamophylax | -0.355 | | 0.269 | -1.317 | 0.192 | -72.983 | | 22.477 | -3.247 | 0.153 |
| Sample_typeGut:TreatmentSericostoma | -1.313 | | 0.276 | -4.757 | **<0.05** | -1.513 | | 22.110 | -0.068 | 0.404 |
| Sample_typeGut:MonthMay | -0.232 | | 0.267 | -0.870 | 0.387 | -27.868 | | 18.066 | -1.543 | 0.379 |
| TreatmentPotamophylax:MonthMay | -0.258 | | 0.305 | -0.845 | 0.401 | -29.102 | | 22.226 | -1.309 | **0.015** |
| TreatmentSericostoma:MonthMay | -0.267 | | 0.305 | -0.875 | 0.385 | 83.710 | | 22.025 | 3.801 | 0.647 |
| Sample_typeGut:TreatmentPotamophylax:MonthMay | 0.198 | | 0.379 | 0.522 | 0.603 | -32.454 | | 31.433 | -1.032 | **0.013** |
| Sample_typeGut:TreatmentSericostoma:MonthMay | -0.038 | | 0.386 | -0.098 | 0.923 | 118.010 | | 31.149 | 3.789 | 0.969 |

***Statistically significant p-values (< 0.05) values are shown in bold***

**Table S5.** Result of the contrast test to observe pairwise comparisons among the sample groups based on marginal mean values of alpha diversity (Shannon and Chao1 indices). The p-values were obtained after applying multivariate *t-*distribution adjustment. Abbreviations; SE: Standard error, df: degrees of freedom.

|  |  |  | **Shannon** | |  | |  |  |  | | **Chao1** | |  | |  | |
| --- | --- | --- | --- | --- | --- | --- | --- | --- | --- | --- | --- | --- | --- | --- | --- | --- |
| **Contrast** | **Estimate** | **SE** | **df** | **t.ratio** | | **p.value** | | **Estimate** | | **SE** | | **df** | | **t.ratio** | | **p.value** |
| Gut Allogamus April - Faeces Allogamus April | -0.286 | 0.190 | 70 | -1.506 | | 0.932 | | -484.481 | | 31.005 | | 70 | | -15.626 | | **<0.001** |
| Gut Allogamus April - Gut Potamophylax April | 0.225 | 0.161 | 70 | 1.400 | | 0.959 | | 30.246 | | 26.204 | | 70 | | 1.154 | | 0.990 |
| Gut Allogamus April - Faeces Potamophylax April | -0.404 | 0.190 | 70 | -2.128 | | 0.600 | | -383.873 | | 31.005 | | 70 | | -12.381 | | **<0.001** |
| Gut Allogamus April - Gut Sericostoma April | 2.219 | 0.172 | 70 | 12.921 | | **<0.001** | | 130.060 | | 28.014 | | 70 | | 4.643 | | 0.001 |
| Gut Allogamus April - Faeces Sericostoma April | 0.639 | 0.190 | 70 | 3.362 | | 0.052 | | -208.454 | | 31.005 | | 70 | | -6.723 | | **<0.001** |
| Gut Allogamus April - Gut Allogamus May | -0.223 | 0.157 | 70 | -1.426 | | 0.953 | | -43.983 | | 25.541 | | 70 | | -1.722 | | 0.849 |
| Gut Allogamus April - Faeces Allogamus May | -0.730 | 0.190 | 70 | -3.838 | | **0.013** | | -589.640 | | 31.005 | | 70 | | -19.017 | | **<0.001** |
| Gut Allogamus April - Gut Potamophylax May | 0.057 | 0.161 | 70 | 0.353 | | 1.000 | | -9.062 | | 26.204 | | 70 | | -0.346 | | 1.000 |
| Gut Allogamus April - Faeces Potamophylax May | -0.592 | 0.190 | 70 | -3.112 | | 0.098 | | -247.504 | | 31.005 | | 70 | | -7.983 | | **<0.001** |
| Gut Allogamus April - Gut Sericostoma May | 2.295 | 0.166 | 70 | 13.863 | | **<0.001** | | 94.780 | | 27.011 | | 70 | | 3.509 | | 0.034 |
| Gut Allogamus April - Faeces Sericostoma May | 0.434 | 0.190 | 70 | 2.281 | | 0.493 | | -240.003 | | 31.005 | | 70 | | -7.741 | | **<0.001** |
| Faeces Allogamus April - Gut Potamophylax April | 0.511 | 0.190 | 70 | 2.690 | | 0.250 | | 514.728 | | 31.005 | | 70 | | 16.601 | | **<0.001** |
| Faeces Allogamus April - Faeces Potamophylax April | -0.118 | 0.216 | 70 | -0.548 | | 1.000 | | 100.608 | | 35.157 | | 70 | | 2.862 | | 0.174 |
| Faeces Allogamus April - Gut Sericostoma April | 2.505 | 0.200 | 70 | 12.555 | | **<0.001** | | 614.542 | | 32.549 | | 70 | | 18.881 | | **<0.001** |
| Faeces Allogamus April - Faeces Sericostoma April | 0.925 | 0.216 | 70 | 4.293 | | **0.003** | | 276.027 | | 35.157 | | 70 | | 7.851 | | **<0.001** |
| Faeces Allogamus April - Gut Allogamus May | 0.063 | 0.187 | 70 | 0.338 | | 1.000 | | 440.499 | | 30.447 | | 70 | | 14.468 | | **<0.001** |
| Faeces Allogamus April - Faeces Allogamus May | -0.443 | 0.216 | 70 | -2.057 | | 0.649 | | -105.158 | | 35.157 | | 70 | | -2.991 | | 0.131 |
| Faeces Allogamus April - Gut Potamophylax May | 0.343 | 0.190 | 70 | 1.804 | | 0.806 | | 475.419 | | 31.005 | | 70 | | 15.333 | | **<0.001** |
| Faeces Allogamus April - Faeces Potamophylax May | -0.305 | 0.216 | 70 | -1.417 | | 0.955 | | 236.978 | | 35.157 | | 70 | | 6.741 | | **<0.001** |
| Faeces Allogamus April - Gut Sericostoma May | 2.582 | 0.194 | 70 | 13.290 | | **<0.001** | | 579.261 | | 31.690 | | 70 | | 18.279 | | **<0.001** |
| Faeces Allogamus April - Faeces Sericostoma May | 0.720 | 0.216 | 70 | 3.340 | | 0.055 | | 244.478 | | 35.157 | | 70 | | 6.954 | | **<0.001** |
| Gut Potamophylax April - Faeces Potamophylax April | -0.629 | 0.190 | 70 | -3.311 | | 0.059 | | -414.119 | | 31.005 | | 70 | | -13.356 | | **<0.001** |
| Gut Potamophylax April - Gut Sericostoma April | 1.994 | 0.172 | 70 | 11.611 | | **<0.001** | | 99.814 | | 28.014 | | 70 | | 3.563 | | **0.029** |
| Gut Potamophylax April - Faeces Sericostoma April | 0.414 | 0.190 | 70 | 2.178 | | 0.565 | | -238.701 | | 31.005 | | 70 | | -7.699 | | **<0.001** |
| Gut Potamophylax April - Gut Allogamus May | -0.448 | 0.157 | 70 | -2.863 | | 0.174 | | -74.229 | | 25.541 | | 70 | | -2.906 | | 0.159 |
| Gut Potamophylax April - Faeces Allogamus May | -0.954 | 0.190 | 70 | -5.022 | | **<0.001** | | -619.886 | | 31.005 | | 70 | | -19.993 | | **<0.001** |
| Gut Potamophylax April - Gut Potamophylax May | -0.168 | 0.161 | 70 | -1.048 | | 0.996 | | -39.309 | | 26.204 | | 70 | | -1.500 | | 0.934 |
| Gut Potamophylax April - Faeces Potamophylax May | -0.817 | 0.190 | 70 | -4.296 | | **0.003** | | -277.750 | | 31.005 | | 70 | | -8.958 | | **<0.001** |
| Gut Potamophylax April - Gut Sericostoma May | 2.070 | 0.166 | 70 | 12.504 | | **<0.001** | | 64.534 | | 27.011 | | 70 | | 2.389 | | 0.422 |
| Gut Potamophylax April - Faeces Sericostoma May | 0.209 | 0.190 | 70 | 1.098 | | 0.994 | | -270.249 | | 31.005 | | 70 | | -8.716 | | **<0.001** |
| Faeces Potamophylax April - Gut Sericostoma April | 2.623 | 0.200 | 70 | 13.147 | | **<0.001** | | 513.934 | | 32.549 | | 70 | | 15.790 | | **<0.001** |
| Faeces Potamophylax April - Faeces Sericostoma April | 1.043 | 0.216 | 70 | 4.842 | | **<0.001** | | 175.419 | | 35.157 | | 70 | | 4.990 | | **<0.001** |
| Faeces Potamophylax April - Gut Allogamus May | 0.181 | 0.187 | 70 | 0.971 | | 0.998 | | 339.890 | | 30.447 | | 70 | | 11.163 | | **<0.001** |
| Faeces Potamophylax April - Faeces Allogamus May | -0.325 | 0.216 | 70 | -1.508 | | 0.931 | | -205.766 | | 35.157 | | 70 | | -5.853 | | **<0.001** |
| Faeces Potamophylax April - Gut Potamophylax May | 0.461 | 0.190 | 70 | 2.426 | | 0.397 | | 374.811 | | 31.005 | | 70 | | 12.089 | | **<0.001** |
| Faeces Potamophylax April - Faeces Potamophylax May | -0.187 | 0.216 | 70 | -0.868 | | 0.999 | | 136.370 | | 35.157 | | 70 | | 3.879 | | **0.012** |
| Faeces Potamophylax April - Gut Sericostoma May | 2.700 | 0.194 | 70 | 13.898 | | **<0.001** | | 478.653 | | 31.690 | | 70 | | 15.104 | | **<0.001** |
| Faeces Potamophylax April - Faeces Sericostoma May | 0.838 | 0.216 | 70 | 3.888 | | **0.011** | | 143.870 | | 35.157 | | 70 | | 4.092 | | **0.006** |
| Gut Sericostoma April - Faeces Sericostoma April | -1.580 | 0.200 | 70 | -7.918 | | **<0.001** | | -338.515 | | 32.549 | | 70 | | -10.400 | | **<0.001** |
| Gut Sericostoma April - Gut Allogamus May | -2.442 | 0.168 | 70 | -14.542 | | **<0.001** | | -174.043 | | 27.394 | | 70 | | -6.353 | | **<0.001** |
| Gut Sericostoma April - Faeces Allogamus May | -2.948 | 0.200 | 70 | -14.776 | | **<0.001** | | -719.700 | | 32.549 | | 70 | | -22.111 | | **<0.001** |
| Gut Sericostoma April - Gut Potamophylax May | -2.162 | 0.172 | 70 | -12.591 | | **<0.001** | | -139.123 | | 28.014 | | 70 | | -4.966 | | **<0.001** |
| Gut Sericostoma April - Faeces Potamophylax May | -2.810 | 0.200 | 70 | -14.085 | | **<0.001** | | -377.564 | | 32.549 | | 70 | | -11.600 | | **<0.001** |
| Gut Sericostoma April - Gut Sericostoma May | 0.077 | 0.176 | 70 | 0.434 | | 1.000 | | -35.280 | | 28.770 | | 70 | | -1.226 | | 0.984 |
| Gut Sericostoma April - Faeces Sericostoma May | -1.785 | 0.200 | 70 | -8.947 | | **<0.001** | | -370.063 | | 32.549 | | 70 | | -11.369 | | **<0.001** |
| Faeces Sericostoma April - Gut Allogamus May | -0.862 | 0.187 | 70 | -4.620 | | **0.001** | | 164.472 | | 30.447 | | 70 | | 5.402 | | **<0.001** |
| Faeces Sericostoma April - Faeces Allogamus May | -1.369 | 0.216 | 70 | -6.350 | | **<0.001** | | -381.185 | | 35.157 | | 70 | | -10.842 | | **<0.001** |
| Faeces Sericostoma April - Gut Potamophylax May | -0.582 | 0.190 | 70 | -3.064 | | 0.111 | | 199.392 | | 31.005 | | 70 | | 6.431 | | **<0.001** |
| Faeces Sericostoma April - Faeces Potamophylax May | -1.231 | 0.216 | 70 | -5.710 | | **<0.001** | | -39.049 | | 35.157 | | 70 | | -1.111 | | 0.993 |
| Faeces Sericostoma April - Gut Sericostoma May | 1.656 | 0.194 | 70 | 8.527 | | **<0.001** | | 303.234 | | 31.690 | | 70 | | 9.569 | | **<0.001** |
| Faeces Sericostoma April - Faeces Sericostoma May | -0.205 | 0.216 | 70 | -0.953 | | 0.998 | | -31.549 | | 35.157 | | 70 | | -0.897 | | 0.999 |
| Gut Allogamus May - Faeces Allogamus May | -0.506 | 0.187 | 70 | -2.712 | | 0.237 | | -545.657 | | 30.447 | | 70 | | -17.922 | | **<0.001** |
| Gut Allogamus May - Gut Potamophylax May | 0.280 | 0.157 | 70 | 1.788 | | 0.816 | | 34.920 | | 25.541 | | 70 | | 1.367 | | 0.965 |
| Gut Allogamus May - Faeces Potamophylax May | -0.368 | 0.187 | 70 | -1.973 | | 0.704 | | -203.521 | | 30.447 | | 70 | | -6.684 | | **<0.001** |
| Gut Allogamus May - Gut Sericostoma May | 2.519 | 0.162 | 70 | 15.582 | | **<0.001** | | 138.763 | | 26.368 | | 70 | | 5.263 | | **<0.001** |
| Gut Allogamus May - Faeces Sericostoma May | 0.657 | 0.187 | 70 | 3.519 | | **<0.001** | | -196.020 | | 30.447 | | 70 | | -6.438 | | **<0.001** |
| Faeces Allogamus May - Gut Potamophylax May | 0.786 | 0.190 | 70 | 4.136 | | **<0.001** | | 580.577 | | 31.005 | | 70 | | 18.725 | | **<0.001** |
| Faeces Allogamus May - Faeces Potamophylax May | 0.138 | 0.216 | 70 | 0.640 | | 1.000 | | 342.136 | | 35.157 | | 70 | | 9.732 | | **<0.001** |
| Faeces Allogamus May - Gut Sericostoma May | 3.025 | 0.194 | 70 | 15.571 | | **<0.001** | | 684.419 | | 31.690 | | 70 | | 21.597 | | **<0.001** |
| Faeces Allogamus May - Faeces Sericostoma May | 1.163 | 0.216 | 70 | 5.397 | | **<0.001** | | 349.637 | | 35.157 | | 70 | | 9.945 | | **<0.001** |
| Gut Potamophylax May - Faeces Potamophylax May | -0.648 | 0.190 | 70 | -3.410 | | **0.045** | | -238.441 | | 31.005 | | 70 | | -7.690 | | **<0.001** |
| Gut Potamophylax May - Gut Sericostoma May | 2.239 | 0.166 | 70 | 13.521 | | **<0.001** | | 103.842 | | 27.011 | | 70 | | 3.844 | | **0.012** |
| Gut Potamophylax May - Faeces Sericostoma May | 0.377 | 0.190 | 70 | 1.983 | | 0.698 | | -230.941 | | 31.005 | | 70 | | -7.448 | | **<0.001** |
| Faeces Potamophylax May - Gut Sericostoma May | 2.887 | 0.194 | 70 | 14.861 | | **<0.001** | | 342.284 | | 31.690 | | 70 | | 10.801 | | **<0.001** |
| Faeces Potamophylax May - Faeces Sericostoma May | 1.025 | 0.216 | 70 | 4.757 | | **0.001** | | 7.501 | | 35.157 | | 70 | | 0.213 | | 1.000 |
| Gut Sericostoma May - Faeces Sericostoma May | -1.862 | 0.194 | 70 | -9.584 | | **<0.001** | | -334.783 | | 31.690 | | 70 | | -10.564 | | **<0.001** |

***Statistically significant p-values (< 0.05) are shown in bold.***


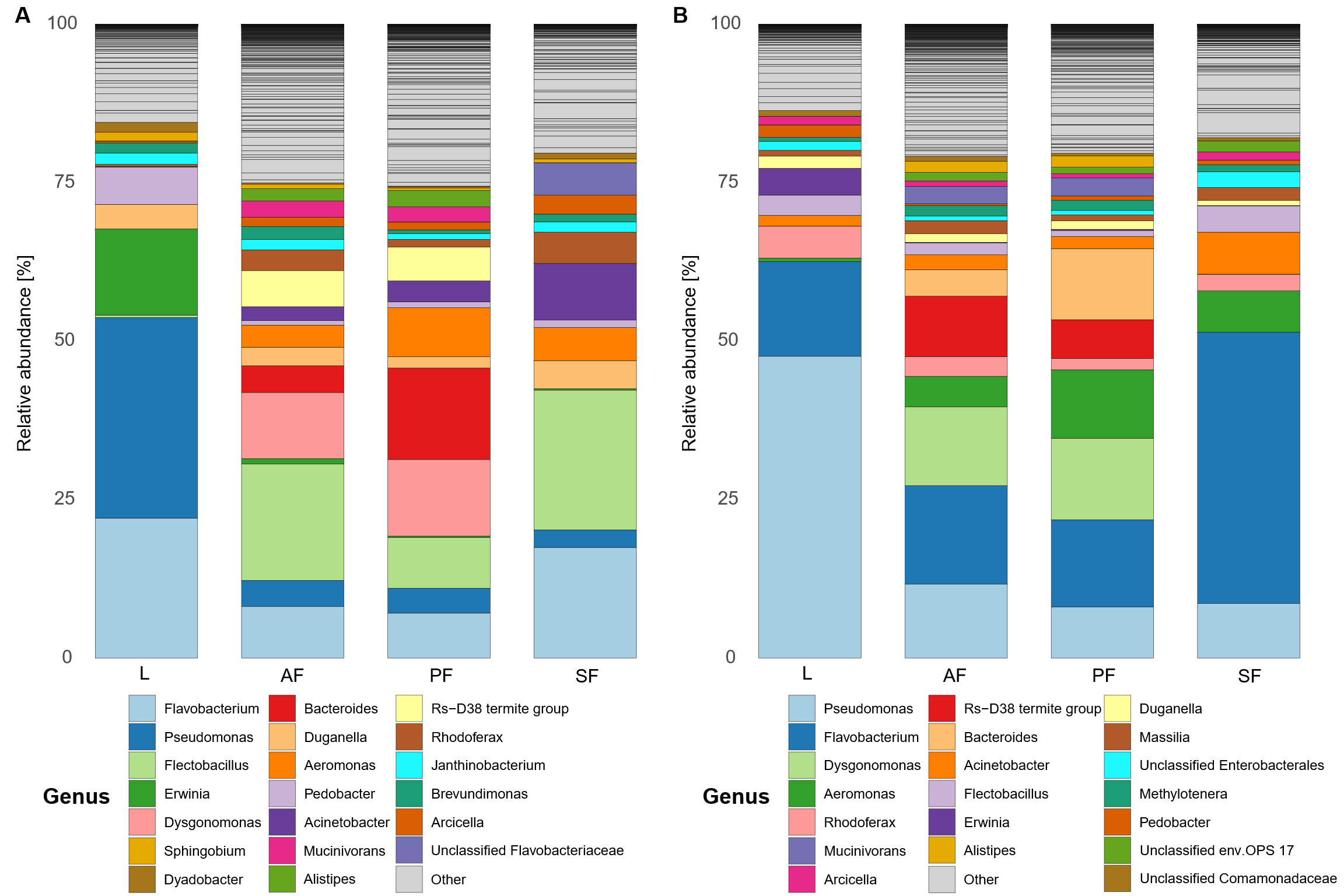


**Figure S1.** Taxonomic composition and relative abundance of individual taxonomic groups associated with leaves, and Faecal pellets produced by each shredder taxon during the feeding experiment in the month of April (A) and May (B) based on complementary DNA (cDNA) dataset. In Fig. each genus is represented by distinct color and top 20 of in relative abundance of bacterial composition at the genus level are shown whereas the remaining order are grouped as “Other”. Abbreviations; L: Leaves, AF: *Allogamus* Faecal pellets, PF: *Potamophylax* Faecal pellets and SF: *Sericostoma* Faecal pellets.


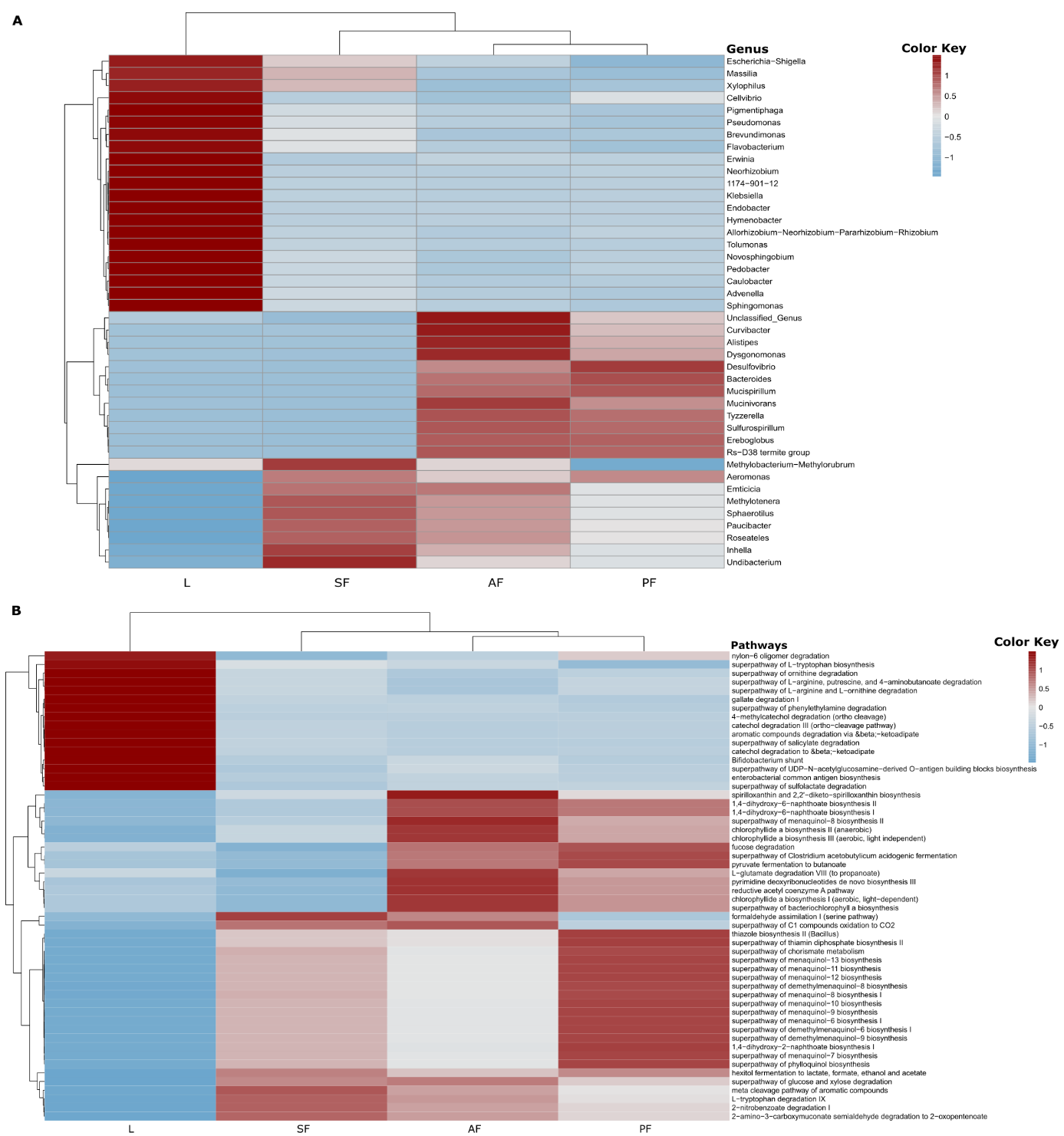


**Figure S2.** Hierarchical heat map of differentially abundant taxa (A) and pathways (B) distributed across leaves, guts and faecal pellets of each shredder taxa are shown. Data used here is cDNA dataset. A hierarchical cluster heat map showing the log2 transformed abundant values for leaves, guts and faecal pellets of each shredder taxa. The differentially abundant individual taxa across the leaves (L) and Faecal pellets of each shredder taxa (AF= *Allogamus* Faecal pellets, PF= *Potamophylax* Faecal pellets, SF= *Sericostoma* Faecal pellets) are shown horizontally. Here color key reflects normalized log-transformed counts. Skyblue shows lower abundance and red color shows a higher abundance.

**Table S6.** Table showing the unique pathways predicted for different sample groups based on whole genomic DNA dataset.

| **Sample_Group** | **Pathway** | **Group_Prevalence** | **Other_Prevalence** | **Description** |
| --- | --- | --- | --- | --- |
| Allogamus gut | PWY-7373 | 1 | 0.48 | superpathway of demethylmenaquinol-6 biosynthesis II |
| Sericostoma gut | GOLPDLCAT-PWY | 0.93 | 0.47 | superpathway of glycerol degradation to 1,3-propanediol |
| Sericostoma gut | PWY-5971 | 1 | 0.47 | palmitate biosynthesis II (bacteria and plants) |
| Sericostoma gut | PWY-6471 | 1 | 0.47 | peptidoglycan biosynthesis IV (Enterococcus faecium) |
| Potamophylax gut | PWY-7373 | 1 | 0.49 | superpathway of demethylmenaquinol-6 biosynthesis II |
| Leaves | PWY-5941 | 1 | 0.43 | glycogen degradation II (eukaryotic) |
| Allogamus Faecal pellets | HCAMHPDEG-PWY | 0.9 | 0.42 | 3-phenylpropanoate and 3-(3-hydroxyphenyl)propanoate degradation to 2-oxopent-4-enoate |
| Allogamus Faecal pellets | P381-PWY | 1 | 0.38 | adenosylcobalamin biosynthesis II (late cobalt incorporation) |
| Allogamus Faecal pellets | PWY-5178 | 0.9 | 0.48 | toluene degradation IV (aerobic) (via catechol) |
| Allogamus Faecal pellets | PWY-5430 | 0.9 | 0.41 | meta cleavage pathway of aromatic compounds |
| Allogamus Faecal pellets | PWY-5507 | 1 | 0.46 | adenosylcobalamin biosynthesis I (early cobalt insertion) |
| Allogamus Faecal pellets | PWY-5531 | 1 | 0.27 | chlorophyllide a biosynthesis II (anaerobic) |
| Allogamus Faecal pellets | PWY-5647 | 1 | 0.4 | 2-nitrobenzoate degradation I |
| Allogamus Faecal pellets | PWY-5654 | 1 | 0.4 | 2-amino-3-carboxymuconate semialdehyde degradation to 2-oxopentenoate |
| Allogamus Faecal pellets | PWY-5655 | 1 | 0.4 | L-tryptophan degradation IX |
| Allogamus Faecal pellets | PWY-5941 | 1 | 0.49 | glycogen degradation II (eukaryotic) |
| Allogamus Faecal pellets | PWY-6690 | 0.9 | 0.42 | cinnamate and 3-hydroxycinnamate degradation to 2-oxopent-4-enoate |
| Allogamus Faecal pellets | PWY-7159 | 1 | 0.27 | chlorophyllide a biosynthesis III (aerobic, light independent) |
| Allogamus Faecal pellets | PWY0-1277 | 0.9 | 0.42 | 3-phenylpropanoate and 3-(3-hydroxyphenyl)propanoate degradation |
| Allogamus Faecal pellets | THREOCAT-PWY | 1 | 0.28 | superpathway of L-threonine metabolism |
| Sericostoma Faecal pellets | HCAMHPDEG-PWY | 1 | 0.41 | 3-phenylpropanoate and 3-(3-hydroxyphenyl)propanoate degradation to 2-oxopent-4-enoate |
| Sericostoma Faecal pellets | PWY-5178 | 1 | 0.47 | toluene degradation IV (aerobic) (via catechol) |
| Sericostoma Faecal pellets | PWY-5180 | 0.9 | 0.35 | toluene degradation I (aerobic) (via o-cresol) |
| Sericostoma Faecal pellets | PWY-5182 | 0.9 | 0.35 | toluene degradation II (aerobic) (via 4-methylcatechol) |
| Sericostoma Faecal pellets | PWY-5183 | 0.9 | 0.18 | superpathway of aerobic toluene degradation |
| Sericostoma Faecal pellets | PWY-5430 | 1 | 0.4 | meta cleavage pathway of aromatic compounds |
| Sericostoma Faecal pellets | PWY-5531 | 0.9 | 0.28 | chlorophyllide a biosynthesis II (anaerobic) |
| Sericostoma Faecal pellets | PWY-5647 | 1 | 0.4 | 2-nitrobenzoate degradation I |
| Sericostoma Faecal pellets | PWY-5654 | 1 | 0.4 | 2-amino-3-carboxymuconate semialdehyde degradation to 2-oxopentenoate |
| Sericostoma Faecal pellets | PWY-5655 | 1 | 0.4 | L-tryptophan degradation IX |
| Sericostoma Faecal pellets | PWY-5941 | 1 | 0.49 | glycogen degradation II (eukaryotic) |
| Sericostoma Faecal pellets | PWY-6690 | 1 | 0.41 | cinnamate and 3-hydroxycinnamate degradation to 2-oxopent-4-enoate |
| Sericostoma Faecal pellets | PWY-7159 | 0.9 | 0.28 | chlorophyllide a biosynthesis III (aerobic, light independent) |
| Sericostoma Faecal pellets | PWY-7391 | 0.9 | 0.15 | isoprene biosynthesis II (engineered) |
| Sericostoma Faecal pellets | PWY0-1277 | 1 | 0.41 | 3-phenylpropanoate and 3-(3-hydroxyphenyl)propanoate degradation |
| Potamophylax Faecal pellets | HCAMHPDEG-PWY | 0.9 | 0.42 | 3-phenylpropanoate and 3-(3-hydroxyphenyl)propanoate degradation to 2-oxopent-4-enoate |
| Potamophylax Faecal pellets | P381-PWY | 1 | 0.38 | adenosylcobalamin biosynthesis II (late cobalt incorporation) |
| Potamophylax Faecal pellets | PWY-5178 | 1 | 0.47 | toluene degradation IV (aerobic) (via catechol) |
| Potamophylax Faecal pellets | PWY-5430 | 1 | 0.4 | meta cleavage pathway of aromatic compounds |
| Potamophylax Faecal pellets | PWY-5507 | 1 | 0.46 | adenosylcobalamin biosynthesis I (early cobalt insertion) |
| Potamophylax Faecal pellets | PWY-5531 | 1 | 0.27 | chlorophyllide a biosynthesis II (anaerobic) |
| Potamophylax Faecal pellets | PWY-5647 | 0.9 | 0.41 | 2-nitrobenzoate degradation I |
| Potamophylax Faecal pellets | PWY-5654 | 0.9 | 0.41 | 2-amino-3-carboxymuconate semialdehyde degradation to 2-oxopentenoate |
| Potamophylax Faecal pellets | PWY-5655 | 0.9 | 0.41 | L-tryptophan degradation IX |
| Potamophylax Faecal pellets | PWY-5941 | 1 | 0.49 | glycogen degradation II (eukaryotic) |
| Potamophylax Faecal pellets | PWY-6690 | 0.9 | 0.42 | cinnamate and 3-hydroxycinnamate degradation to 2-oxopent-4-enoate |
| Potamophylax Faecal pellets | PWY-7159 | 1 | 0.27 | chlorophyllide a biosynthesis III (aerobic, light independent) |
| Potamophylax Faecal pellets | PWY0-1277 | 0.9 | 0.42 | 3-phenylpropanoate and 3-(3-hydroxyphenyl)propanoate degradation |
| Potamophylax Faecal pellets | THREOCAT-PWY | 1 | 0.28 | superpathway of L-threonine metabolism |

**Table S7.** Summary of the differential pathways identified using DESeq2 analysis. All the significant pathways with a log fold change > 2.5 were listed here. baseMean corresponds to the mean of normalized counts for each pathway across the samples. log2FoldChange (log2FC) represents the estimated log2-scale effect size for the comparison across the samples. abs_logFC is the absolute effect size and reflects the magnitude of change regardless of direction. IfcSE represents standard error of the log2 Fold Change. Data used here was whole genomic DNA dataset.

| **Pathway ID** | **Description** | **baseMean** | **log2FoldChange** | **abs_log2FoldChange** | **lfcSE** | **stat** |
| --- | --- | --- | --- | --- | --- | --- |
| PWY-6182 | superpathway of salicylate degradation | 606.63 | -9.81 | 9.81 | 0.28 | -35.14 |
| PWY-6895 | superpathway of thiamin diphosphate biosynthesis II | 2332.60 | 11.62 | 11.62 | 0.34 | 34.42 |
| PWY-5417 | catechol degradation III (ortho-cleavage pathway) | 647.79 | -9.97 | 9.97 | 0.29 | -33.86 |
| PWY-5431 | aromatic compounds degradation via &beta;-ketoadipate | 647.79 | -9.97 | 9.97 | 0.29 | -33.86 |
| CATECHOL-ORTHO-CLEAVAGE-PWY | catechol degradation to &beta;-ketoadipate | 593.11 | -9.98 | 9.98 | 0.30 | -33.69 |
| PWY-6185 | 4-methylcatechol degradation (ortho cleavage) | 606.47 | -10.10 | 10.10 | 0.30 | -33.13 |
| PWY-5181 | toluene degradation III (aerobic) (via p-cresol) | 768.35 | -9.83 | 9.83 | 0.30 | -32.27 |
| PWY0-321 | phenylacetate degradation I (aerobic) | 808.74 | -9.96 | 9.96 | 0.33 | -30.05 |
| DHGLUCONATE-PYR-CAT-PWY | glucose degradation (oxidative) | 777.84 | -9.48 | 9.48 | 0.32 | -29.68 |
| PWY-6071 | superpathway of phenylethylamine degradation | 697.87 | -10.20 | 10.20 | 0.35 | -29.18 |
| PWY-5104 | L-isoleucine biosynthesis IV | 4849.53 | 3.48 | 3.48 | 0.12 | 29.14 |
| PWY-7431 | aromatic biogenic amine degradation (bacteria) | 1707.13 | -8.98 | 8.98 | 0.31 | -29.12 |
| ALL-CHORISMATE-PWY | superpathway of chorismate metabolism | 1299.71 | 10.31 | 10.31 | 0.36 | 28.72 |
| PWY-5838 | superpathway of menaquinol-8 biosynthesis I | 1769.15 | 10.59 | 10.59 | 0.37 | 28.54 |
| PWY-5840 | superpathway of menaquinol-7 biosynthesis | 1534.09 | 10.50 | 10.50 | 0.37 | 28.11 |
| PWY-5897 | superpathway of menaquinol-11 biosynthesis | 1646.83 | 10.45 | 10.45 | 0.37 | 27.90 |
| PWY-5898 | superpathway of menaquinol-12 biosynthesis | 1646.83 | 10.45 | 10.45 | 0.37 | 27.90 |
| PWY-5899 | superpathway of menaquinol-13 biosynthesis | 1646.83 | 10.45 | 10.45 | 0.37 | 27.90 |
| PROTOCATECHUATE-ORTHO-CLEAVAGE-PWY | protocatechuate degradation II (ortho-cleavage pathway) | 1167.44 | -8.63 | 8.63 | 0.32 | -27.25 |
| PWY-6545 | pyrimidine deoxyribonucleotides de novo biosynthesis III | 570.18 | 10.39 | 10.39 | 0.38 | 27.25 |
| PWY-5845 | superpathway of menaquinol-9 biosynthesis | 1497.49 | 10.28 | 10.28 | 0.38 | 27.13 |
| PWY-5850 | superpathway of menaquinol-6 biosynthesis I | 1497.49 | 10.28 | 10.28 | 0.38 | 27.13 |
| PWY-5896 | superpathway of menaquinol-10 biosynthesis | 1497.49 | 10.28 | 10.28 | 0.38 | 27.13 |
| GALLATE-DEGRADATION-II-PWY | gallate degradation I | 444.60 | -9.75 | 9.75 | 0.36 | -26.95 |
| PWY-5861 | superpathway of demethylmenaquinol-8 biosynthesis | 1394.93 | 10.13 | 10.13 | 0.38 | 26.56 |
| PWY-6891 | thiazole biosynthesis II (Bacillus) | 890.80 | 9.86 | 9.86 | 0.37 | 26.39 |
| PWY-6478 | GDP-D-glycero-&alpha;-D-manno-heptose biosynthesis | 852.72 | 11.11 | 11.11 | 0.43 | 25.54 |
| PWY-7376 | cob(II)yrinate a,c-diamide biosynthesis II (late cobalt incorporation) | 679.65 | -8.71 | 8.71 | 0.35 | -25.22 |
| PWY-5860 | superpathway of demethylmenaquinol-6 biosynthesis I | 1157.85 | 9.80 | 9.80 | 0.39 | 25.15 |
| PWY-5862 | superpathway of demethylmenaquinol-9 biosynthesis | 1157.85 | 9.80 | 9.80 | 0.39 | 25.15 |
| P42-PWY | incomplete reductive TCA cycle | 4763.83 | 3.38 | 3.39 | 0.14 | 24.85 |
| PWY-6396 | superpathway of 2,3-butanediol biosynthesis | 119.87 | -8.32 | 8.32 | 0.35 | -23.77 |
| P125-PWY | superpathway of (R,R)-butanediol biosynthesis | 89.26 | -8.06 | 8.06 | 0.34 | -23.48 |
| PWY-5863 | superpathway of phylloquinol biosynthesis | 892.71 | 9.30 | 9.31 | 0.40 | 23.40 |
| PWY-5837 | 1,4-dihydroxy-2-naphthoate biosynthesis I | 822.73 | 9.13 | 9.13 | 0.40 | 22.68 |
| CODH-PWY | reductive acetyl coenzyme A pathway | 125.90 | 8.61 | 8.62 | 0.38 | 22.46 |
| PWY-6590 | superpathway of Clostridium acetobutylicum acidogenic fermentation | 385.38 | 9.59 | 9.59 | 0.43 | 22.14 |
| PWY-7373 | superpathway of demethylmenaquinol-6 biosynthesis II | 120.04 | 8.74 | 8.74 | 0.40 | 21.96 |
| CENTFERM-PWY | pyruvate fermentation to butanoate | 313.51 | 9.27 | 9.27 | 0.42 | 21.94 |
| PWY-6901 | superpathway of glucose and xylose degradation | 1461.17 | 7.67 | 7.67 | 0.36 | 21.10 |
| PWY-7254 | TCA cycle VII (acetate-producers) | 3859.53 | -3.51 | 3.51 | 0.17 | -20.45 |
| PWY-6338 | superpathway of vanillin and vanillate degradation | 149.66 | -7.07 | 7.07 | 0.35 | -20.36 |
| PWY-7097 | vanillin and vanillate degradation I | 149.66 | -7.07 | 7.07 | 0.35 | -20.36 |
| PWY-7098 | vanillin and vanillate degradation II | 162.22 | -7.10 | 7.10 | 0.35 | -20.13 |
| RUMP-PWY | formaldehyde oxidation I | 756.86 | -6.24 | 6.24 | 0.32 | -19.72 |
| P441-PWY | superpathway of N-acetylneuraminate degradation | 367.09 | 10.77 | 10.77 | 0.55 | 19.65 |
| FUC-RHAMCAT-PWY | superpathway of fucose and rhamnose degradation | 163.90 | 7.71 | 7.71 | 0.40 | 19.36 |
| PWY-2941 | L-lysine biosynthesis II | 349.64 | 10.82 | 10.82 | 0.56 | 19.22 |
| PWY-5705 | allantoin degradation to glyoxylate III | 162.09 | -8.17 | 8.17 | 0.43 | -19.11 |
| PWY-1861 | formaldehyde assimilation II (RuMP Cycle) | 1137.35 | -6.26 | 6.27 | 0.33 | -18.73 |
| FASYN-INITIAL-PWY | superpathway of fatty acid biosynthesis initiation (E. coli) | 4603.44 | -2.53 | 2.53 | 0.14 | -18.57 |
| P562-PWY | myo-inositol degradation I | 362.90 | -5.62 | 5.62 | 0.31 | -18.03 |
| FUCCAT-PWY | fucose degradation | 92.57 | 7.34 | 7.34 | 0.41 | 18.01 |
| PWY-7003 | glycerol degradation to butanol | 214.52 | 8.13 | 8.13 | 0.45 | 17.96 |
| GLCMANNANAUT-PWY | superpathway of N-acetylglucosamine, N-acetylmannosamine and N-acetylneuraminate degradation | 169.17 | 9.73 | 9.73 | 0.55 | 17.78 |
| PWY-7007 | methyl ketone biosynthesis | 320.61 | -6.81 | 6.81 | 0.39 | -17.57 |
| DENITRIFICATION-PWY | nitrate reduction I (denitrification) | 82.37 | -7.35 | 7.35 | 0.42 | -17.50 |
| ARGORNPROST-PWY | arginine, ornithine and proline interconversion | 995.29 | -3.17 | 3.17 | 0.18 | -17.28 |
| PWY-181 | photorespiration | 378.30 | -7.38 | 7.38 | 0.43 | -17.28 |
| PWY0-1338 | polymyxin resistance | 279.39 | -5.18 | 5.18 | 0.31 | -16.98 |
| PWY-5651 | L-tryptophan degradation to 2-amino-3-carboxymuconate semialdehyde | 1437.39 | -5.60 | 5.60 | 0.33 | -16.95 |
| P461-PWY | hexitol fermentation to lactate, formate, ethanol and acetate | 270.95 | 6.24 | 6.24 | 0.37 | 16.94 |
| PWY-5022 | 4-aminobutanoate degradation V | 1411.24 | -3.36 | 3.36 | 0.20 | -16.79 |
| NAD-BIOSYNTHESIS-II | NAD salvage pathway II | 768.49 | -4.39 | 4.39 | 0.26 | -16.78 |
| PWY490-3 | nitrate reduction VI (assimilatory) | 677.46 | -6.09 | 6.09 | 0.37 | -16.68 |
| PWY-5920 | superpathway of heme biosynthesis from glycine | 1218.76 | 3.07 | 3.07 | 0.18 | 16.67 |
| PWY-7237 | myo-, chiro- and scillo-inositol degradation | 1087.26 | -5.95 | 5.95 | 0.36 | -16.56 |
| PWY-5100 | pyruvate fermentation to acetate and lactate II | 3529.41 | 4.60 | 4.60 | 0.28 | 16.47 |
| NADSYN-PWY | NAD biosynthesis II (from tryptophan) | 1107.82 | -5.17 | 5.17 | 0.31 | -16.47 |
| PWY0-1533 | methylphosphonate degradation I | 351.26 | -4.73 | 4.73 | 0.29 | -16.34 |
| PWY-7090 | UDP-2,3-diacetamido-2,3-dideoxy-&alpha;-D-mannuronate biosynthesis | 24.58 | -5.83 | 5.84 | 0.36 | -16.31 |
| AST-PWY | L-arginine degradation II (AST pathway) | 1263.12 | -4.03 | 4.03 | 0.25 | -16.06 |
| PWY-6263 | superpathway of menaquinol-8 biosynthesis II | 647.46 | 6.62 | 6.62 | 0.42 | 15.71 |
| PWY-7371 | 1,4-dihydroxy-6-naphthoate biosynthesis II | 452.06 | 3.48 | 3.48 | 0.22 | 15.59 |
| PWY-7374 | 1,4-dihydroxy-6-naphthoate biosynthesis I | 434.45 | 3.42 | 3.42 | 0.22 | 15.43 |
| PWY-5941 | glycogen degradation II (eukaryotic) | 30.57 | -5.94 | 5.94 | 0.39 | -15.26 |
| P221-PWY | octane oxidation | 1180.55 | -2.73 | 2.73 | 0.19 | -14.76 |
| ORNDEG-PWY | superpathway of ornithine degradation | 874.87 | -7.09 | 7.09 | 0.49 | -14.53 |
| GALLATE-DEGRADATION-I-PWY | gallate degradation II | 535.02 | -3.11 | 3.11 | 0.22 | -14.36 |
| PWY-722 | nicotinate degradation I | 16.96 | -5.35 | 5.35 | 0.37 | -14.34 |
| PWY-5747 | 2-methylcitrate cycle II | 1285.58 | -3.38 | 3.38 | 0.24 | -14.21 |
| PWY-1361 | benzoyl-CoA degradation I (aerobic) | 95.80 | -5.67 | 5.67 | 0.40 | -14.19 |
| PWY-6588 | pyruvate fermentation to acetone | 116.12 | 6.94 | 6.94 | 0.50 | 13.93 |
| PWY-5028 | L-histidine degradation II | 1335.10 | -3.41 | 3.41 | 0.25 | -13.61 |
| METHYLGALLATE-DEGRADATION-PWY | methylgallate degradation | 594.15 | -2.91 | 2.91 | 0.22 | -13.49 |
| ARGDEG-PWY | superpathway of L-arginine, putrescine, and 4-aminobutanoate degradation | 420.49 | -6.39 | 6.39 | 0.49 | -13.00 |
| ORNARGDEG-PWY | superpathway of L-arginine and L-ornithine degradation | 420.49 | -6.39 | 6.39 | 0.49 | -13.00 |
| PWY-7377 | cob(II)yrinate a,c-diamide biosynthesis I (early cobalt insertion) | 94.03 | 6.09 | 6.09 | 0.48 | 12.60 |
| PWY-6562 | norspermidine biosynthesis | 597.89 | -5.43 | 5.43 | 0.45 | -12.13 |
| P124-PWY | Bifidobacterium shunt | 437.07 | -5.68 | 5.68 | 0.47 | -12.06 |
| SALVADEHYPOX-PWY | adenosine nucleotides degradation II | 2631.11 | -2.80 | 2.80 | 0.23 | -11.95 |
| PWY-5304 | superpathway of sulfur oxidation (Acidianus ambivalens) | 877.51 | 2.86 | 2.86 | 0.26 | 11.08 |
| ECASYN-PWY | enterobacterial common antigen biosynthesis | 239.26 | -3.22 | 3.22 | 0.30 | -10.73 |
| PWY-6339 | syringate degradation | 623.83 | -6.02 | 6.02 | 0.60 | -10.11 |
| PWY-5910 | superpathway of geranylgeranyldiphosphate biosynthesis I (via mevalonate) | 55.24 | -4.05 | 4.05 | 0.41 | -9.92 |
| METHGLYUT-PWY | superpathway of methylglyoxal degradation | 70.84 | -5.62 | 5.62 | 0.57 | -9.89 |
| PWY-5507 | adenosylcobalamin biosynthesis I (early cobalt insertion) | 65.15 | 5.16 | 5.16 | 0.53 | 9.75 |
| P101-PWY | ectoine biosynthesis | 90.80 | 2.58 | 2.59 | 0.27 | 9.66 |
| PWY-922 | mevalonate pathway I | 40.50 | -4.05 | 4.05 | 0.42 | -9.59 |
| PWY-7295 | L-arabinose degradation IV | 53.42 | -3.81 | 3.81 | 0.41 | -9.36 |
| PWY-5971 | palmitate biosynthesis II (bacteria and plants) | 500.26 | 5.79 | 5.79 | 0.69 | 8.34 |
| LIPASYN-PWY | phospholipases | 30.10 | 4.52 | 4.52 | 0.56 | 8.12 |
| P23-PWY | reductive TCA cycle I | 2651.90 | 2.73 | 2.73 | 0.35 | 7.77 |
| P381-PWY | adenosylcobalamin biosynthesis II (late cobalt incorporation) | 70.15 | 3.12 | 3.13 | 0.54 | 5.83 |
| PWY0-1277 | 3-phenylpropanoate and 3-(3-hydroxyphenyl)propanoate degradation | 13.26 | -2.66 | 2.66 | 0.46 | -5.72 |
| PWY-5178 | toluene degradation IV (aerobic) (via catechol) | 14.61 | -2.70 | 2.70 | 0.48 | -5.56 |
| PWY-6471 | peptidoglycan biosynthesis IV (Enterococcus faecium) | 66.26 | 2.86 | 2.86 | 0.59 | 4.83 |

**Table S8.** Summary of the differential pathways identified using DESeq2 analysis. All the significant pathways with a log fold change > 2 were listed here. *baseMean* corresponds to the mean of normalized counts for each pathway across the samples. log2FoldChange (log2FC) represents the estimated log2-scale effect size for the comparison across the samples. abs_logFC is the absolute effect size and reflects the magnitude of change regardless of direction. IfcSE represents standard error of the log2 Fold Change. Data used here was cDNA dataset.

| **Pathway ID** | **Description** | **baseMean** | **log2FoldChange** | **abs_log2FC** | **lfcSE** | **stat** |
| --- | --- | --- | --- | --- | --- | --- |
| PWY-6545 | pyrimidine deoxyribonucleotides de novo biosynthesis III | 43.94 | -7.76 | 7.76 | 0.41 | -19.03 |
| CODH-PWY | reductive acetyl coenzyme A pathway | 26.03 | -6.66 | 6.66 | 0.40 | -16.59 |
| PWY-7371 | 1,4-dihydroxy-6-naphthoate biosynthesis II | 62.27 | -3.52 | 3.52 | 0.23 | -15.21 |
| PWY-7374 | 1,4-dihydroxy-6-naphthoate biosynthesis I | 62.07 | -3.52 | 3.52 | 0.23 | -15.18 |
| CHLOROPHYLL-SYN | chlorophyllide a biosynthesis I (aerobic, light-dependent) | 56.17 | -6.47 | 6.47 | 0.44 | -14.69 |
| CENTFERM-PWY | pyruvate fermentation to butanoate | 199.81 | -6.88 | 6.88 | 0.48 | -14.48 |
| PWY-6590 | superpathway of Clostridium acetobutylicum acidogenic fermentation | 240.31 | -6.91 | 6.91 | 0.50 | -13.74 |
| PWY-5529 | superpathway of bacteriochlorophyll a biosynthesis | 67.19 | -6.22 | 6.22 | 0.48 | -12.96 |
| PWY-6182 | superpathway of salicylate degradation | 693.30 | 2.17 | 2.17 | 0.18 | 12.29 |
| PWY-6185 | 4-methylcatechol degradation (ortho cleavage) | 720.03 | 2.35 | 2.35 | 0.19 | 12.16 |
| PWY-5417 | catechol degradation III (ortho-cleavage pathway) | 707.14 | 2.28 | 2.28 | 0.19 | 12.08 |
| PWY-5431 | aromatic compounds degradation via &beta;-ketoadipate | 707.14 | 2.28 | 2.28 | 0.19 | 12.08 |
| CATECHOL-ORTHO-CLEAVAGE-PWY | catechol degradation to &beta;-ketoadipate | 652.44 | 2.25 | 2.25 | 0.19 | 11.82 |
| GALLATE-DEGRADATION-II-PWY | gallate degradation I | 523.98 | 2.80 | 2.80 | 0.25 | 11.21 |
| PWY-6071 | superpathway of phenylethylamine degradation | 796.60 | 2.39 | 2.39 | 0.22 | 10.85 |
| PWY-5531 | chlorophyllide a biosynthesis II (anaerobic) | 91.44 | -4.53 | 4.53 | 0.47 | -9.67 |
| PWY-7159 | chlorophyllide a biosynthesis III (aerobic, light independent) | 91.44 | -4.53 | 4.53 | 0.47 | -9.67 |
| PWY-6581 | spirilloxanthin and 2,2'-diketo-spirilloxanthin biosynthesis | 43.69 | -4.24 | 4.24 | 0.45 | -9.53 |
| P124-PWY | Bifidobacterium shunt | 275.16 | 2.50 | 2.50 | 0.27 | 9.17 |
| ECASYN-PWY | enterobacterial common antigen biosynthesis | 100.36 | 4.28 | 4.28 | 0.47 | 9.14 |
| PWY-6901 | superpathway of glucose and xylose degradation | 655.94 | -3.46 | 3.46 | 0.39 | -8.91 |
| P461-PWY | hexitol fermentation to lactate, formate, ethanol and acetate | 172.32 | -2.10 | 2.10 | 0.25 | -8.54 |
| PWY-5088 | L-glutamate degradation VIII (to propanoate) | 14.17 | -4.11 | 4.11 | 0.50 | -8.27 |
| PWY-6891 | thiazole biosynthesis II (Bacillus) | 348.42 | -3.11 | 3.11 | 0.38 | -8.09 |
| PWY-1622 | formaldehyde assimilation I (serine pathway) | 105.83 | -5.58 | 5.58 | 0.69 | -8.06 |
| ARGDEG-PWY | superpathway of L-arginine, putrescine, and 4-aminobutanoate degradation | 244.65 | 2.66 | 2.66 | 0.34 | 7.87 |
| ORNARGDEG-PWY | superpathway of L-arginine and L-ornithine degradation | 244.65 | 2.66 | 2.66 | 0.34 | 7.87 |
| PWY-5840 | superpathway of menaquinol-7 biosynthesis | 596.25 | -2.59 | 2.59 | 0.33 | -7.80 |
| PWY-5430 | meta cleavage pathway of aromatic compounds | 68.89 | -2.95 | 2.95 | 0.38 | -7.75 |
| PWY-5654 | 2-amino-3-carboxymuconate semialdehyde degradation to 2-oxopentenoate | 33.93 | -2.95 | 2.95 | 0.38 | -7.71 |
| PWY-5647 | 2-nitrobenzoate degradation I | 38.61 | -2.95 | 2.95 | 0.39 | -7.65 |
| PWY-5861 | superpathway of demethylmenaquinol-8 biosynthesis | 544.80 | -2.64 | 2.64 | 0.35 | -7.63 |
| PWY-5860 | superpathway of demethylmenaquinol-6 biosynthesis I | 454.13 | -2.72 | 2.72 | 0.36 | -7.58 |
| PWY-5862 | superpathway of demethylmenaquinol-9 biosynthesis | 454.13 | -2.72 | 2.72 | 0.36 | -7.58 |
| ORNDEG-PWY | superpathway of ornithine degradation | 916.25 | 2.47 | 2.47 | 0.33 | 7.46 |
| PWY-5838 | superpathway of menaquinol-8 biosynthesis I | 702.28 | -2.52 | 2.52 | 0.34 | -7.44 |
| PWY-5897 | superpathway of menaquinol-11 biosynthesis | 655.84 | -2.54 | 2.54 | 0.34 | -7.42 |
| PWY-5898 | superpathway of menaquinol-12 biosynthesis | 655.84 | -2.54 | 2.54 | 0.34 | -7.42 |
| PWY-5899 | superpathway of menaquinol-13 biosynthesis | 655.84 | -2.54 | 2.54 | 0.34 | -7.42 |
| PWY-5655 | L-tryptophan degradation IX | 66.81 | -2.83 | 2.83 | 0.38 | -7.41 |
| PWY-5845 | superpathway of menaquinol-9 biosynthesis | 595.45 | -2.61 | 2.61 | 0.35 | -7.37 |
| PWY-5850 | superpathway of menaquinol-6 biosynthesis I | 595.45 | -2.61 | 2.61 | 0.35 | -7.37 |
| PWY-5896 | superpathway of menaquinol-10 biosynthesis | 595.45 | -2.61 | 2.61 | 0.35 | -7.37 |
| PWY-5837 | 1,4-dihydroxy-2-naphthoate biosynthesis I | 340.81 | -2.68 | 2.68 | 0.37 | -7.25 |
| PWY-5863 | superpathway of phylloquinol biosynthesis | 369.64 | -2.65 | 2.65 | 0.37 | -7.21 |
| FUCCAT-PWY | fucose degradation | 95.79 | -2.88 | 2.88 | 0.40 | -7.13 |
| PWY-7332 | superpathway of UDP-N-acetylglucosamine-derived O-antigen building blocks biosynthesis | 162.00 | 2.22 | 2.22 | 0.33 | 6.68 |
| PWY-6263 | superpathway of menaquinol-8 biosynthesis II | 65.50 | -3.11 | 3.11 | 0.48 | -6.52 |
| PWY-6895 | superpathway of thiamin diphosphate biosynthesis II | 978.25 | -2.52 | 2.52 | 0.39 | -6.46 |
| ALL-CHORISMATE-PWY | superpathway of chorismate metabolism | 694.62 | -2.20 | 2.20 | 0.35 | -6.34 |
| PWY-1882 | superpathway of C1 compounds oxidation to CO2 | 47.05 | -2.91 | 2.91 | 0.47 | -6.20 |
| PWY-6641 | superpathway of sulfolactate degradation | 29.49 | 2.03 | 2.03 | 0.48 | 4.21 |
| P621-PWY | nylon-6 oligomer degradation | 18.16 | 2.17 | 2.17 | 0.54 | 4.04 |
| PWY-6629 | superpathway of L-tryptophan biosynthesis | 112.23 | 2.17 | 2.17 | 0.57 | 3.81 |
